# Cryo-EM structure determination of small therapeutic protein targets at 3 Å-resolution using a rigid imaging scaffold

**DOI:** 10.1073/pnas.2305494120

**Published:** 2023-09-05

**Authors:** Roger Castells-Graells, Kyle Meador, Mark A. Arbing, Michael R. Sawaya, Morgan Gee, Duilio Cascio, Emma Gleave, Judit É. Debreczeni, Jason Breed, Karoline Leopold, Ankoor Patel, Dushyant Jahagirdar, Bronwyn Lyons, Sriram Subramaniam, Chris Phillips, Todd O. Yeates

**Affiliations:** ^a^Department of Energy, Institute for Genomics and Proteomics, University of California, Los Angeles, CA 90095; ^b^Department of Chemistry and Biochemistry, University of California, Los Angeles, CA 90095; ^c^Discovery Sciences, R&D, AstraZeneca, Cambridge CB2 0AA, United Kingdom; ^d^Gandeeva Therapeutics, Inc., Burnaby, British Columbia V5C 6N5, Canada; ^e^Department of Biochemistry and Molecular Biology, The University of British Columbia, Vancouver, BC V6T 1Z3, Canada

**Keywords:** cryo-EM, small proteins, imaging scaffolds, protein design, cancer drugs

## Abstract

Cryoelectron microscopy (Cryo-EM) has enabled structural determination of proteins larger than about 50 kDa, including many intractable by any other method, but it has largely failed for smaller proteins. Here, we obtain structures of small proteins by binding them to a rigid molecular scaffold based on a designed protein cage, revealing atomic details at resolutions reaching 2.9 Å. We apply this system to the key cancer signaling protein KRAS (19 kDa in size), obtaining four structures of oncogenic mutational variants by cryo-EM. Importantly, a structure for the key G12C mutant bound to an inhibitor drug (AMG510) reveals significant conformational differences compared to prior data in the crystalline state. The findings highlight the promise of cryo-EM scaffolds for advancing the design of drug molecules against small therapeutic protein targets in cancer and other human diseases.

## Significance

Cryoelectron microscopy (cryo-EM) is emerging as a major method for elucidating the structures of proteins in atomic detail. A key limitation, however, is that cryo-EM is applicable only to sufficiently large macromolecular complexes. This places a great many important proteins of smaller size, especially those of interest for therapeutic drug development, outside the reach of cryo-EM. We describe a protein engineering effort that overcomes the lower mass limit through the development of a modular imaging scaffold able to rigidly bind and display practically any small protein of interest, greatly increasing its effective mass. We show this technology can be used to visualize molecules, such as a key cancer protein, with important implications for drug design and biomedical research.

Cryoelectron microscopy (cryo-EM) is a rapidly expanding method for determining the atomic structures of large molecular assemblies. It is, however, problematic for determining the structures of small-to-medium-sized protein molecules. A size of about 38 kDa represents a likely theoretical lower limit (1), while about 50 kDa is a practical limit from current work (2). Accordingly, vast numbers of cellular proteins, including many of key therapeutic interest, remain beyond the reach of cryo-EM methods (3).

A potential workaround to the size limitation in cryo-EM is to bind a small protein of interest (the “cargo”) to a much larger carrier (the “scaffold”) in order to make it large enough to visualize readily. Ideas for scaffolding approaches go back several years (4–6). A key challenge is how to make the binding attachment between the scaffold and the cargo protein sufficiently rigid, as even minor flexibility in the attachment severely compromises the ability to reconstruct a high-resolution image of the bound cargo component. In addition, a general solution to the scaffolding problem calls for modular design, i.e., through the use of a scaffolding component that can be readily diversified to bind any given cargo protein of interest (7–10). Earlier work has explored the use of DARPins as the modular binding domain, genetically fused by way of a continuous alpha helical connection to self-assembling protein cages, to create large symmetric scaffolds for imaging (11–14). Diverse studies have made progress (2, 15–20) (*SI Appendix*, *Supplementary Text*), but further improvements are needed to develop a facile system for high-resolution cryo-EM of small proteins.

In the present study, we demonstrate a protein design advance that substantially rigidifies a cryo-EM scaffold based on fusion of a DARPin as the modular binding domain to a designed protein cage. Analogous to antibodies, sequence variations in the nonconserved loop regions of a DARPin protein can be selected in the laboratory in order to obtain a variant that binds nearly any protein of interest (21). To demonstrate utility in a critically important area of medicine, we have applied this rigidified cryo-EM scaffolding system to study mutant and drug-bound structures of the key oncogenic protein KRAS, which represents a major target for designing anticancer drugs.

## Results and Discussion

### Rigidification and Testing of an Imaging Scaffold.

**Fig. 1.**
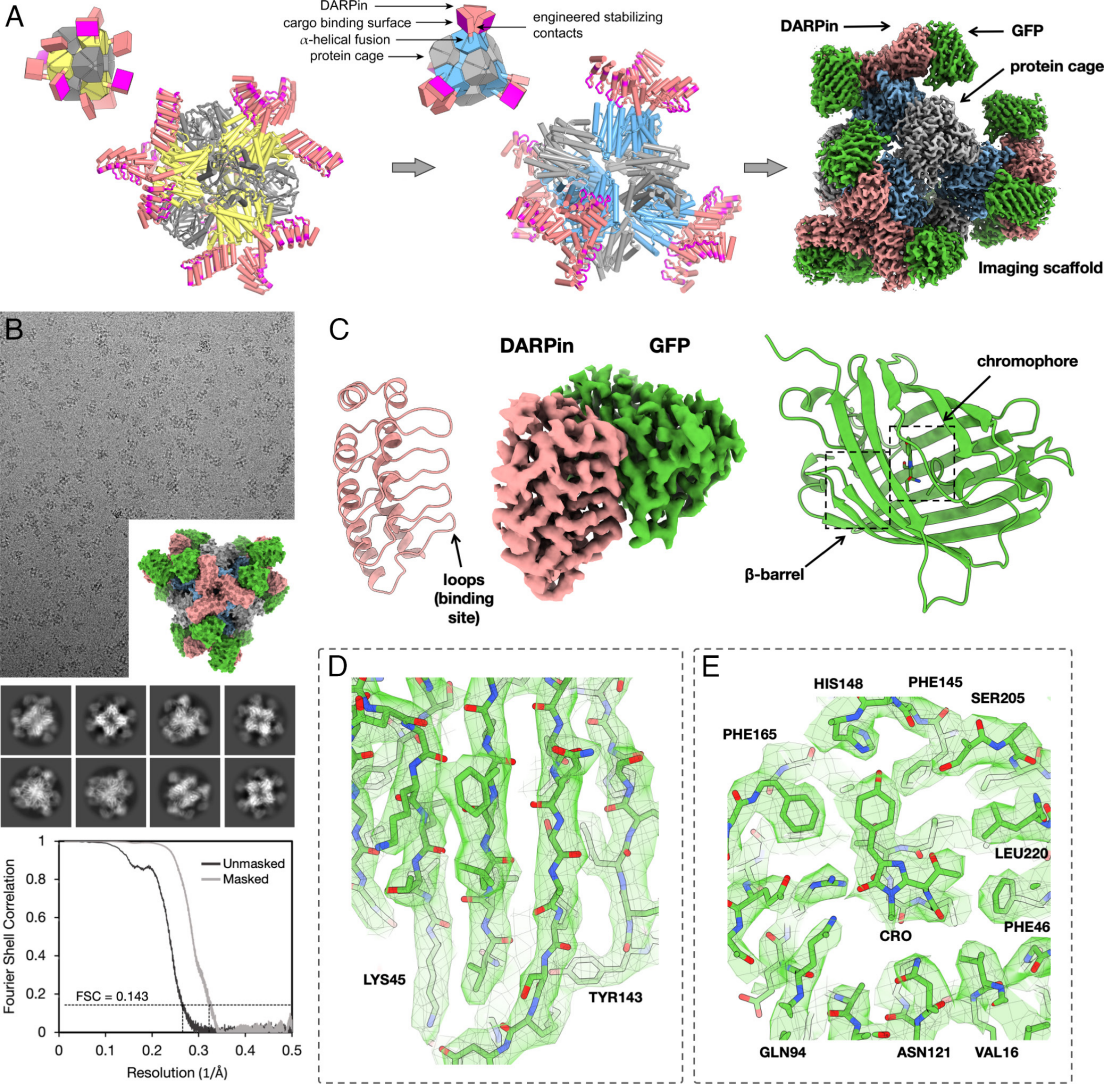
Rigidified modular cryo-EM imaging scaffolds. (*A*, *Left*) A scheme for a previously described scaffold (11, 12), based on a self-assembling protein cage, displayed protruding DARPin domains as modular binders via continuous alpha helical fusions. The cage subunits bearing the continuous alpha helical fusion are shown in yellow. The other subunit type in this two-component cage is shown in gray. DARPin domains are colored in salmon with their hypervariable binding regions highlighted in magenta. (*A*, *Middle*) A redesigned scaffold based on similar principles, but with protruding DARPin arms disposed to make additional protein–protein contacts with symmetric copies of each other. Designed surface mutations at the newly created interface away from the hypervariable region stabilize the DARPin domain, allowing high-resolution cryo-EM imaging of bound cargo. The *Insets* provide simplified geometric diagrams of the scaffold constructions. (*A*, *Right*) Composite cryo-EM map after focused refinements of GFP bound to a rigidified imaging scaffold. (*B*) Cryo-EM micrograph of the rigidified imaging scaffold bound to GFP (model shown in *Inset*) and 2D classes from selected particles. An FSC plot illustrates agreement between independent half-maps obtained after focused classification and 3D reconstruction, masked around the GFP protein (resolution = 3.1 Å based on a correlation threshold of 0.143). (*C*, *Middle*) A view of the final density map covering the DARPin and its bound GFP protein. Ribbon models of the two components are shown on the sides. (*D* and *E*) Focused views of the density map covering several GFP beta-strands and the GFP chromophore with its surrounding amino acid side chains.

A previous cage-scaffold design reached a resolution of about 3.8 Å for the attached cargo protein (11, 12), but residual flexibility made it impossible to reach the higher resolution needed for reliable atomic interpretation (generally about 3 Å or better). In the earlier design, the individual DARPin arms—12 in total emanating from the tetrahedrally symmetric cage—protruded separately from each other, thus suffering from residual flexibility. To make further stabilizing contacts possible, we investigated alternative design choices for a scaffold. A different tetrahedral protein cage known as T33-51 (22), when modeled with alpha helical linkers to DARPins, oriented the protruding arms to be in near-contact with each other; three DARPins come together at each of the four vertices of the tetrahedron (Fig. 1). Then, computational interface design methods were used to generate new amino acid sequences at the interfaces formed between three symmetry-related copies of the DARPin (*SI Appendix*, Fig. S1 and *Materials and Methods*). The designed interfaces between protruding DARPins were proposed to confer additional stability to these key binding components of the scaffold (Fig. 1). From 12 candidate sequence designs, five were validated by experimental tests to self-assemble into cage-like structures as intended (*Materials and Methods*).

Before employing the candidate cryo-EM scaffolds to image a protein target of major biological importance, we compared their performance in a test system, using the well-studied superfolder version of the green fluorescent protein (GFP) (23), 26 kDa in size, as the cargo protein. When bound to the imaging scaffold, the overall molecular weight of this complex is 972 kDa. As expected, experimental tests showed that all five scaffold candidates bound to GFP when the DARPin (genetically fused to the cage) was one previously established to bind GFP (*SI Appendix*). Initial cryo-EM datasets were collected on the five candidate scaffolds with GFP bound. Based on data processing of similar numbers of particle images from the five candidates, one design (designated RCG-10; *SI Appendix*) appeared to offer the most rigid presentation of the bound GFP cargo protein. This scaffold was therefore selected for further analysis and cryo-EM data processing. Following data processing from ~877,000 particles obtained from 3,575 cryo-EM movies, a 3-D density map was obtained in which the resolution of the central core of the scaffold was 2.7 Å, with a resolution of 3.1 Å for just the GFP component (Fig. 1 and *SI Appendix*, Figs. S4 and S5). The level of atomic detail is illustrated by the density for the GFP chromophore and side chains from the neighboring amino acid residues (Fig. 1).

In order to assess issues related to coordinate precision and potential perturbances caused by binding to the scaffold, we compared the bound protein structure to crystal structures of GFP in an unbound form. The binding of GFP to the DARPin did not lead to meaningful differences in the backbone, though a different rotamer is seen for a tyrosine residue (Tyr39). The rms deviation for the GFP displayed by the imaging scaffold compared to a crystal structure is 0.59 Å. For data quality and model refinement statistics, see *SI Appendix*, Table S1.

While the significant improvement in resolution of the cargo (compared to the previous, unrigidified scaffold) also reflects various advances in cryo-EM instrumentation and software, analysis of the data shows that the scaffold redesign did lead to a dramatic reduction in the flexibility of the cargo attachment, as anticipated (*SI Appendix*, Fig. S12). The success of the rigidification plan is evident in the pattern of agreement between the atomic model and the cryo-EM density map; the agreement Q-scores decrease steeply with distance from the core-DARPin hinge in the old design but remain nearly uniform in the new design (*SI Appendix*, Fig. S12). Importantly, this supports the hinge as a principal cause of reduced resolution of the cargo in the old design and the reduction in hinge flexibility as a major cause of improvement in the new design.

Additionally, we compared the ability of the deep-learning program ModelAngelo (24) to build de novo atomic models into the cryo-EM density maps. For the earlier 3.8-Å cryo-EM map, the program correctly built only 93 residues (including sidechain atoms) of 156 DARPin residues, a roughly 60% completion for the DARPin. Only 65 of 231 residues could be built for the GFP cargo, corresponding to only 28% completeness. For the new 3.1-Å cryo-EM map, ModelAngelo built all 156 residues of the DARPin domain correctly (100% success), including sidechains. For the GFP cargo, the program built 220 of 231 residues correctly (95% success), including sidechains. The missing residues are in loops (*SI Appendix*, Fig. S13).

### Cryo-EM Structures of the Oncogenic KRAS Protein Bound to GDP.

For biomedically relevant structural studies, we chose the KRAS protein as a target of high clinical importance. KRAS is a 19-kDa GTPase involved in signal transduction in cell proliferation pathways. KRAS is among the most prevalent human oncogenes, with mutations in KRAS occurring in about 25% of all cancers (25). Some of the most clinically relevant mutations occur at amino acid residues Gly12 and Gly13. Drugs bound to a minor cleft region of the protein near that location are of key pharmaceutical interest, including covalent inhibitors targeting cysteine mutants (i.e., G12C or G13C) (26–29). We therefore undertook a series of structural studies on known KRAS mutants, focusing on the degree of atomic interpretability in 3D density maps obtained using the cryo-EM scaffold described above; a DARPin with loop sequences that bind the GDP-bound form of KRAS was already known from prior work (30, 31), enabling the scaffold to be readily repurposed to image GDP-bound KRAS structures (*Materials and Methods*).

**Fig. 2.**
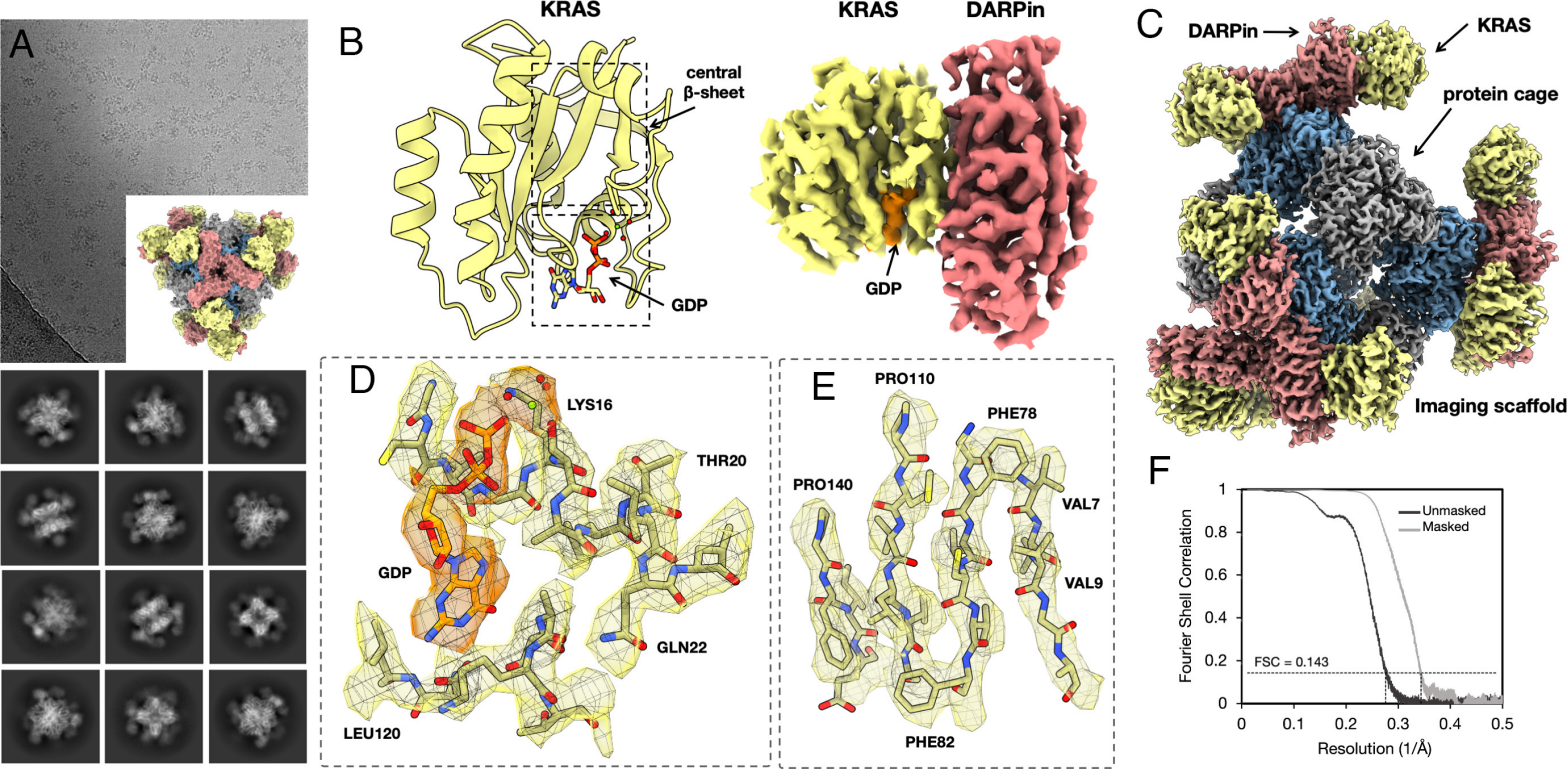
Cryo-EM structure of KRAS on a rigidified imaging scaffold. (*A*) Cryo-EM micrograph of the rigidified imaging scaffold bound to KRAS (model shown in *Inset*) and 2D classes from the selected particles. (*B*) 3D reconstruction of a density map covering the DARPin and its bound KRAS protein. The GDP ligand is shown in orange. A ribbon model of the KRAS is shown on the left side. (*C*) Composite cryo-EM map after focused refinements of KRAS bound to a rigidified imaging scaffold. (*D* and *E*) Focused views of the density map covering the bound GDP ligand (orange density) and select regions of the KRAS structure. The Mg^2+^ ion is represented by a green sphere. (*F*) An FSC plot illustrates agreement between independent half-maps, obtained after focused classification and 3D reconstruction, masked around the KRAS protein (resolution = 2.9 Å based on a correlation threshold of 0.143).

For imaging experiments, we investigated three different sequence variants of KRAS—single site mutants G12V, G12C, and G13C—in their GDP-bound forms. All three KRAS variants were found to bind with good occupancy to our cryo-EM scaffold (presenting the KRAS-specific DARPin). For mutant G13C, ~665,000 particles were obtained from 2,000 cryo-EM movies. Following similar data processing as before, we obtained a 3-D density map showing a resolution of 2.5 Å for the entire particle and 2.9 Å for the KRAS protein (Fig. 2 and *SI Appendix*, Figs. S7 and S8). Among other metrics of map quality, we assessed the ability of automatic protein model-building software to generate an atomic model for the protein without human intervention. Given the cryo-EM density map and the amino acid sequences for the DARPin and KRAS proteins, ModelAngelo (24) was able to build, de novo, a correct and nearly complete atomic model using default parameters (164 out of 166 residues for KRAS and 150 out of 157 for the DARPin). The amino acid sequence was correctly assigned throughout both KRAS G13C and DARPin molecules. Limited manual fitting was sufficient to join breaks in the chain where the density was weak for mobile loops in the proteins. The success of the modeling exercise shows the utility of the cryo-EM scaffolding approach for an automated structure determination pipeline.

**Fig. 3.**
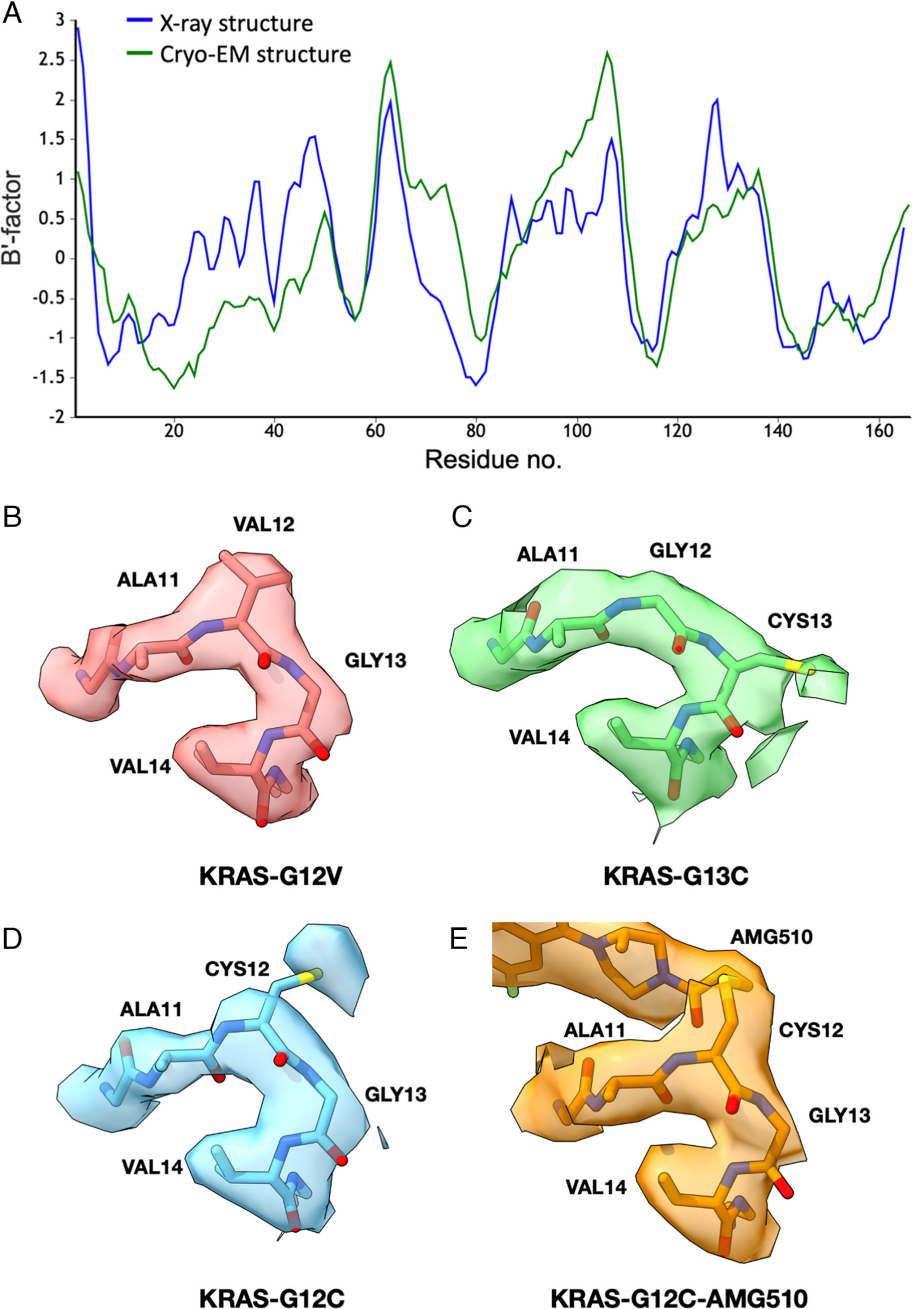
Structural and dynamical interpretability of cryo-EM maps of KRAS and single-site mutants. (*A*) A plot of refined B-factors—a measure of flexibility or dynamic mobility—for the KRAS structure. Agreement is evident between the X-ray crystal structure (pdb 5o2s) and the cryo-EM structure, which was built and refined de novo (after setting B-factors to a uniform starting value of 20 Å^2^). The B-factors are averaged over individual amino acid residues and smoothed over a three-residue window, then normalized for direct comparison using the BANΔIT toolkit (32). The calculated correlation coefficient is 0.65. (*B*–*E*) Cryo-EM density maps around the single site mutations for KRAS G12V, G13C, G12C, and G12C bound to AMG510. A higher-than-average mobility of Cys12 is also reported by X-ray crystallography (pdb6oim).

As imaged here by cryo-EM, the KRAS protein matches closely to known structures of KRAS-GDP reported in previous X-ray crystallography studies (30, 31). Our refined structure of the G13C mutant overlaps with a previous X-ray crystal structure with an rms deviation of only 0.5 Å over protein backbone atoms. The region around the bound GDP cofactor further emphasizes the atomic interpretability (Fig. 2). A Mg^2+^ ion bound near the terminal GDP phosphate group is also clearly visible. An interpretation of protein flexibility and dynamics from the cryo-EM map also agreed well with prior data, as revealed by an analysis of B-factors (or atomic displacement parameters). When examined across the length of the KRAS protein sequence, the correlation coefficient was 0.65 for the atomic structure obtained by cryo-EM compared to an earlier structure reported by X-ray crystallography (Fig. 3*A*). This highlights that the resolution and map quality obtained by cryo-EM are high enough to provide detailed atomic interpretation as well as potentially important information about conformational flexibility.

Structures of additional KRAS mutants provided further opportunities to evaluate atomic interpretability. Following similar protocols as for the G13C mutant, for the G12V mutant, we obtained a final map reconstruction with a resolution of 2.4 Å for the entire particle and 3.1 Å around the KRAS protein (*Materials and Methods*). For the G12C mutant, the resolution was 2.2 Å for the entire particle and 3.0 Å around the KRAS protein (*Materials and Methods*). The maps and refined KRAS structures were all closely comparable, with significant differences in the maps occurring only at the mutated amino acid side chains, as anticipated (Fig. 3). As an assessment of coordinate precision, the rms deviation between the two most closely related cryo-EM structures (the G12V and G12C mutants) was 0.58 Å; this is slightly less than the differences when compared to previously reported X-ray crystal structure, which are between 0.73 and 1.1 Å (*SI Appendix*, Table S2).

### Conformational Variations and Drug Binding to KRAS G12C.

A minor or “cryptic” cleft in the KRAS protein around residues 12 and 13 has been a site of intense focus for drug design efforts (27–29). Substantial protein conformational changes occur in that region upon drug binding; energetic and structural differences caused by drug binding stabilize the KRAS protein in its inactive form, which binds preferentially to GDP. Understanding the conformational and energetic landscape of the KRAS protein in this binding cleft region is expected to advance the discovery of new cancer drugs. Among drugs targeting clinically important KRAS mutations are a subset that form covalent bonds to cysteine mutants in that site.

**Fig. 4.**
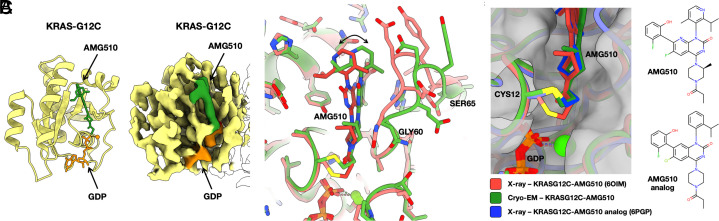
Cryo-EM structure of KRAS G12C bound to AMG510. (*A*) A refined atomic model (*A*, *Left*) and a cryo-EM density map (*A*, *Right*) covering the KRAS protein, with the AMG510 drug molecule bound. The GDP ligand is shown in orange, and the AMG510 drug is in green. (*B*) Comparison between the cryo-EM structure and a prior X-ray crystal structure of KRAS G12C bound to AMG510. (*C*) Conformational variation at the covalent bond between Cys12 and the AMG510 and an AMG510 analog in X-ray and cryo-EM structures. At the thioether attachment, the cryo-EM model resembles an X-ray crystal structure of a complex with an AMG510 analog.

As a test of our cryo-EM scaffold for analyzing KRAS drug binding, we determined the structure of the KRAS G12C mutant bound to the covalent inhibitor drug AMG510 [also known as sotorasib; (33)]. Following similar data processing protocols as before, from a set of 69,949 particle images obtained from 2072 cryo-EM movies, we obtained a density map with a resolution of 2.2 Å for the entire particle and 3.2 Å around the KRAS protein bound to AMG510. The map revealed significant conformation changes in the KRAS G12C mutant protein upon binding the AMG510 inhibitor compared to the G12C structure without drug bound. This was anticipated based on prior X-ray crystal structures showing conformational changes in this key region upon drug binding (28, 34–37). Most notable, however, is that the AMG510-bound structure we obtained by cryo-EM differs in the drug-binding region from the structure of the same complex reported earlier by X-ray crystallography protein structure database (PDB 6oim). The nominal resolution in the cryo-EM map is lower than that reported for the X-ray crystal structure (1.65 Å) (33), but the density is sufficiently well resolved to derive a conformation for bound AMG510 that is different from that observed in the crystallographic structure (*SI Appendix*, Fig. S9), especially at the covalent attachment point (residue 12) and the loop residues 60-GQEEYSAM-67 (Fig. 4). The torsion angle at the covalent bond between Cys12 and the drug molecule AMG510 differs by about 100° in the cryo-EM model from the conformation reported in a crystallographic model of the same drug complex (Fig. 4). A movement of ~ 2.7 Å is evident in regions of the drug molecule around the isopropyl pyridyl group, distal from the point of covalent attachment to Cys 12. We assessed the confidence in our modeling of the AMG510 drug molecule in a test in which we refined atomic models separately into density maps produced using two independent half-datasets. For the drug molecule, the differences between the independent models were only 0.1 to 0.3 Å. This is considerably smaller than the coordinate differences observed in comparison to the reported X-ray structure, which exceeded 2 Å, supporting the conclusion that meaningful differences are being revealed between the reported X-ray and cryo-EM conformations for drug binding (*SI Appendix*, Fig. S14).

Motivated by differences observed in the drug-binding pocket of the KRAS G12C mutant, we surveyed the PDB for examples of KRAS G12C bound to other inhibitors or drug molecules. An analysis of a set of 12 such structures (pdb 7a47, 6pgp, 6pgo, 8dnj, 8dnk, 8dni, 7a1y, 5v9o, 5v9l, 4lv6, 4luc, and 4lyh), all elucidated by X-ray crystallography, highlights a substantial degree of conformational variability for the KRAS protein in the binding region. Some of this variation is clearly the result of differences in the chemical structures of the various bound drugs. But there are unexpected patterns. Interestingly, whereas the cryo-EM structure reported here for the AMG510 drug complex differs from a prior X-ray crystal structure of the identical complex (as discussed above), it matches more closely to an alternative X-ray crystal structure of a complex with a slightly different AMG510 analog (Fig. 4*C*). In particular, we note that the covalent attachment geometry for AMG 510 derived by cryo-EM occurs as well in the context of different drug bound complexes of KRAS G12C.

The findings on AMG510 binding suggest a substantial range of apparently low-energy conformations for the drug molecules and surrounding segments of the protein. The particular conformation observed appears to be affected at least in part by other molecular interactions. In the X-ray crystal structure, the drug-binding region (residues 62 to 73) is at a crystal packing interface (*SI Appendix*, Fig. S15*A*); conformational changes imposed by crystallographic molecular packing have long been studied and proven useful in uncovering conformational states involved in molecular function such as catalysis (38). Likewise, it is notable that in the cryo-EM structure, residue Met 67 is in contact with one of the DARPin domains protruding from the scaffold (*SI Appendix*, Fig. S15*B*). The observed variation across structures provides potentially useful insight into the conformational landscape for drug binding.

## Conclusions

These initial structural findings serve as a starting point for deeper explorations of KRAS, and other small therapeutic protein targets, by cryo-EM scaffolding methods. Two immediate messages emerge. The first concerns feasibility. The rigidified scaffold described here provides a number of advantageous properties for cryo-EM structure determination—size, symmetry, and modular binding—making it suitable for future applications to many important systems. Second, the observation of conformational variability in drug binding emphasizes that cryo-EM approaches are likely to offer alternative structural views and distinct atomic frameworks for drug design efforts across broad areas of medicine.

## Materials and Methods

### Conformational Sampling of Rigidified Scaffolds.

The N-terminal helix of DARP14-3G124Mut5 (12) was spatially aligned to the C-terminal helix of each subunit from the T33-51 cage (22). Using local programs, superpositions were performed between the first five helical residues of the DARPin to five residue windows from the terminal helical region of the protein cage, with different choices for the alignment segment from the protein cage. Following superposition, each conformation was evaluated for detrimental, overlapping collisions, and potentially favorable contacts in the fully assembled symmetric environment using local programs as well as visual inspection. Promising conformations—those where multiple protruding DARPin arms came into close proximity—were subjected to further conformational exploration by allowing for minor helix flexing. Modeling of allowable deviations from ideal alpha helix geometry was based on natural deviations observed in a large set of alpha helices extracted from high-resolution crystal structures.

### Interface Design Calculation.

All calculations were performed in the context of tetrahedral symmetry. For each sampled alignment and helical bend conformation, the resulting pose was relaxed into the REF2015 score function (39) using the FastRelax mover (40). Then, residues in the aligned helical fusion as well as any residues located in cage subunits or other DARPins (excluding variable loop regions) within 8 Å of the aligned DARPin were marked as designable. Further, all residues within 8 Å of designable residues were designated as packable. Sequence design trajectories were performed with a coordinate constraint applied to backbone atoms using Rosetta FastDesign with the InterfaceDesign2019 protocol (41) and REF2015 score function. We collected interface design metrics to quantify the resulting design success as compared to native interfaces (42). After analysis of the global design pool, we removed entire poses from consideration where the average design trajectory had a measured shape complementarity below 0.6, leaving eight viable poses for sampling sequence variations. Next, we ranked the design trajectories from each passing pose by applying a linear weighting scheme to the normalized metrics from each pose. These consisted of favoring fewer buried unsatisfied hydrogen bonds, lower interface energy (between complexed and unbound forms), higher interface shape complementarity, and lower interface solvation energy. Each normalized metric was equally weighted and summed to rank each trajectory. Finally, by examining the sequence diversity of the top candidates from each pose, we removed redundant sequence mutation patterns and selected 12 individual designs for characterization.

### Protein Production.

The sequences of the imaging scaffolds used in this paper are listed below. DNA fragments carrying the designed imaging scaffold sequences were synthesized (Integrated DNA Technologies and Twist Bioscience) and separately cloned into the vectors pET-22b (subunitB-DARPin) or pSAM (subunitA) (gifted from Jumi Shin, Addgene plasmid #45174; http://n2t.net/addgene:45174; RRID:Addgene_45174). The superfolder GFP V206A (sfGFP V206A) vector was previously described (12). DNA manipulations were carried out in *Escherichia coli* XL2 cells (Agilent). The proteins were expressed in *E. coli* BL21(DE3) cells (New England Biolabs) in Terrific Broth at 18 °C overnight with 0.5 mM IPTG induction at an OD_600_ of 1.0.

Upon collection of the cells, pellets were resuspended in buffer (50 mM Tris, 300 mM NaCl, 20 mM imidazole, pH 8.0) supplemented with benzonase nuclease, 1 mM PMSF, EDTA-free protease inhibitor cocktail (Thermo Scientific) and 0.1% LDAO and lysed using an EmulsiFlex C3 homogenizer (Avestin). The cell lysate was cleared by centrifugation at 20,000 × g for 20 min at 4 °C; the resulting supernatant was recovered and centrifuged at 10,000 × g for 10 min at 4 °C and then loaded onto a HisTrap column (GE Healthcare) pre-equilibrated with the same resuspension buffer. The imaging scaffold was eluted with a linear gradient to 300 mM imidazole. Upon elution, 5 mM EDTA and 5 mM BME were added immediately for designs 5, 8, 10, 13, and 14. The eluted proteins were concentrated using Amicon Ultra-15 100-kDa molecular weight cutoff for the imaging scaffold and 3-kDa molecular weight cutoff for the GFP protein. The concentrated proteins were further purified by size exclusion chromatography using a Superose six Increase column, eluted with 20 mM Tris pH 8.0, 100 mM NaCl, 5 mM BME, 5 mM EDTA for designs 5, 8, 10, 13, and 14 and 20 mM Tris, pH 8.0, and 100 mM NaCl for design 33. Chromatography fractions were analyzed by SDS-PAGE and negative stain EM for the presence of the imaging scaffold. KRAS G12V and KRAS G13C proteins were prepared as previously described by Kettle et al. (43).

The DNA sequence encoding wild-type KRAS (1 to 169) was synthesized (Genscript) and cloned into a pET28 vector with an N-terminal 6xHis tag followed by a TEV site. The G12C mutation was introduced using site-directed mutagenesis and confirmed by sequencing. Protein was expressed in BL21(DE3) cells in LB at 16 °C overnight, following induction at OD_600_ of 0.7 with 0.5 mM IPTG. After harvesting, cell pellets were resuspended in purification buffer (20 mM HEPES, pH 7.4, 300 mM NaCl, 0.5 mM TCEP, and 5 mM MgCl_2_) supplemented with 1x EDTA-free protease inhibitor cocktail and 400 units benzonase and lysed by sonication. Cleared lysate was loaded onto a 1-mL HisTrap column (Cytiva), washed with 20 CV purification buffer +25 mM Imidazole, and eluted using an imidazole gradient to 500 mM Imidazole. Peak fractions were pooled, concentrated, and loaded onto a Superdex 75 Increase size-exclusion column in SEC buffer (purification buffer excluding MgCl_2_). For AMG510-bound protein, KRAS G12C was incubated with AMG510 at a 2:1 molar ratio for 30 min and subjected to size-exclusion chromatography (Superose 6 Increase). Peak fractions yielded a mixture of AMG510-bound and free KRAS G12C (see *SI Appendix*, Fig. S10, first lane).

Either KRAS G12C or KRAS G12C-AMG510 was mixed with the imaging scaffold at a 2:1 molar ratio, incubated on ice for 5 min, and complex formation was confirmed through size-exclusion chromatography (Superose 6 Increase).

### Negative Stain EM.

The concentration of a 3.5-µL sample of fresh Superose six Increase eluent was adjusted to ~100 µg/mL, applied to glow-discharged Formvar/Carbon 400 mesh Cu grids (Ted Pella Inc) for 1 min and blotted to remove excess liquid. After a wash with filtered MilliQ water, the grid was stained with 2% uranyl acetate for 1 min. Images were taken on a Tecnai T12, a T20, a TF20, and a Talos F200C.

### Cryo-EM Data Collection.

Concentrated imaging scaffolds (1 to 10 mg/mL) were mixed with the GFP cargo or KRAS G13C/KRAS G12V/ KRAS G12C/KRAS G12C-AMG510 to a molar ratio of 1:2 and diluted to a final concentration of 0.5 to 0.7 mg/mL. The final buffer composition was 20 mM Tris, pH 8.0, and 100 mM NaCl.

Quantifoil 300 mesh R2/2 copper grids were glow discharged for 30 s at 15 mA using a PELCO easiGLow (Ted Pella). A 1.8- to 3.5-µL volume of sample was applied to the grid at a temperature of 10 or 18 °C at ~100% relative humidity, followed by blotting and vitrification into liquid ethane using a Vitrobot Mark IV Thermo Fisher Scientific. Cryo-EM data were collected on an FEI Titan Krios cryoelectron microscope equipped with a Gatan K3 Summit direct electron detector and on a Titan Krios G4 cryoelectron microscope (Thermo Fisher Scientific) equipped with a Falcon4 direct electron detector in electron event registration mode. With the Gatan K3 Summit detector, movies were recorded with Leginon (44) and SerialEM (45) at a nominal magnification of 81,000× (calibrated pixel size of 1.1 Å per pixel) for designs 5, 8, 10, 13, 14, 33 (G13C) datasets and at a nominal magnification of 105,000× (calibrated pixel size of 0.856 Å per pixel) for design 33 (G12V) dataset, over a defocus range of −1.0 to −2.2 µm. With the Falcon4 detector, movies were recorded with the EPU automated acquisition software at a nominal magnification of 155,000× (calibrated pixel size of 0.5 Å per pixel), for design 33 (G12C and G12C-AMG510) datasets, over a target defocus range of −1.00 µm to −2.25 µm with increment steps of 0.25 µm and a total dose of 40 e^−^/Å^2^.

Fourier shell correlation (FSC) calculations are summarized in *SI Appendix*, Fig. S11. Plots showing dependence of resolution on the number of particles are shown in *SI Appendix*, Fig. S16.

### Cryo-EM Data Processing and Model Building.

Motion correction, CTF estimation, particle picking, 2D classification, and further data processing were performed with cryoSPARC v.3.2 (46). An initial set of particles was automatically picked using a blob-picker protocol. The extracted particles were 2D classified after which an ab initio reconstruction was generated. This reconstruction was then used for the 3D refinements enforcing T symmetry. The 3D structure was used to generate 2D projections of the particles and then used to repick the particles from the images using a template picker. The picked particles were extracted from the micrographs and went through 3D refinements enforcing T symmetry. The symmetry was then expanded, followed by further focused 3D classification without alignments and focused refinements using a mask encompassing the density for one DARPin and one cargo protein, GFP or KRAS, respectively. The best-resolved classes from the focused 3D classification were focused refined (C1 symmetry) performing local angular searches with the fulcrum at the center of mass of the mask. For the GFP imaging scaffold, we obtained an overall resolution of 2.7 Å for the entire particle and a resolution of 3.1 Å over the GFP protein, based on an FSC threshold of 0.143. For the KRAS G13C imaging scaffold, we obtained an overall resolution of 2.5 Å for the entire particle, and the resolution over the KRAS protein was 2.9 Å. We performed automatic de novo atomic model building into our KRAS G13C cryo-EM density using the program ModelAngelo (24) in the COSMIC^2^ platform (47). The structure of GFP was built de novo using the automated chain tracing program, Buccaneer (48). The other three structures reported here were built starting from atomic models of close homologs, as noted in *SI Appendix*, Table S1. Manual adjustments to the models were performed using Coot (49), and automated refinement was performed using Phenix (50). Figures were prepared using ChimeraX (51, 52) and PyMOL (Version 2.0 Schrödinger, LLC)

### Refinement into Half-Maps.

We used refinement against independent half-maps (reconstructed from independent half-datasets) as an assessment of coordinate precision for the bound AMG510 drug molecule. Prior to independent real-space refinement, the molecules were subjected to computational simulated annealing—heating to 1,000 K and slow cooling to 300 K—in the program Phenix.

### FSC Calculation.

FSC plots were generated using the *mtriage* tool of Phenix (53). Each refined model and final map were submitted to *mtriage* along with two half-maps. Masked curves correspond to the use of a smoothed mask to perform FSC calculation only around the model (54).

### Retrospective Test of Scaffold Structure Predictability by AI Methods.

Given the important interplay between protein sequence design and protein structure prediction, we considered whether a leading machine learning algorithm, AlphaFold2 (55), would correctly predict the structure of our designed scaffold based on amino acid sequence. Such a success would argue that an unguided algorithm might have reached the same (or a similar) design result. A key element of the present scaffold design is the association of a homomeric protein trimer—based on a protein chain comprising a cage subunit fused to a DARPin—in such a fashion that stabilizing interactions occur between three copies of the DARPin; the trimer is mainly held together by association of the cage subunit component. When applied to our designed protein sequence, and specifying three chains to be associated, the AlphaFold2 program did not faithfully recapitulate the key stabilizing features between DARPins that were critical in rigidifying the scaffold to enable high-resolution imaging, and which were validated by cryo-EM. For example, residue ARG 254 was engineered to make a stabilizing interaction with residue ASP 181 from an adjacent DARPin. In our cryo-EM structure, those two residues come into atomic contact, as intended. In contrast, prediction by AlphaFold2 leaves those two residues ~15 Å apart, which is well beyond interaction distance. We furthermore attempted to use AlphaFold2 to computationally assemble the entire 24 subunit (a_12_b_12_) scaffold architecture given just the amino acid sequence information. That computational exercise did not assemble the cage subunits into a correct tetrahedral assembly. These results emphasize the importance in the present work of expert human input in the overall design strategy.

## Supplementary Material

Appendix 01 (PDF)

## Data Availability

The structures of the imaging scaffolds and the protein targets, and their associated atomic coordinates, have been deposited into the Electron Microscopy Data Bank (EMDB) and the Protein Data Bank (PDB) with EMDB accession codes EMD-29700 (56), EMD-29713 (57), EMD-29715 (58), EMD-29718 (59), EMD-29719 (60), and EMD-29720 (61) and PDB accession codes 8G3K (62), 8G42 (63), 8G47 (64), 8G4E (65), 8G4F (66), and 8G4H (67), respectively. The sequences of the protein designs are included in *SI Appendix*.
