# Supplementary material for: Cryo-EM structure determination of small therapeutic protein targets at 3 Å-resolution using a rigid imaging scaffold
Source: Proc Natl Acad Sci U S A. 2023 Sep 5;120(37):e2305494120. doi: 10.1073/pnas.2305494120 (PMC10500258; doi:10.1073/pnas.2305494120)
Supplement: Supplementary file 1 — Appendix 01 (PDF) [file pnas.2305494120.sapp.pdf]

Supplementary Material for

Castells-Graells, *et al.*
"Cryo-EM structure determination of small therapeutic protein targets at 3 Å resolution using a rigid imaging scaffold"

Supplementary Figures:

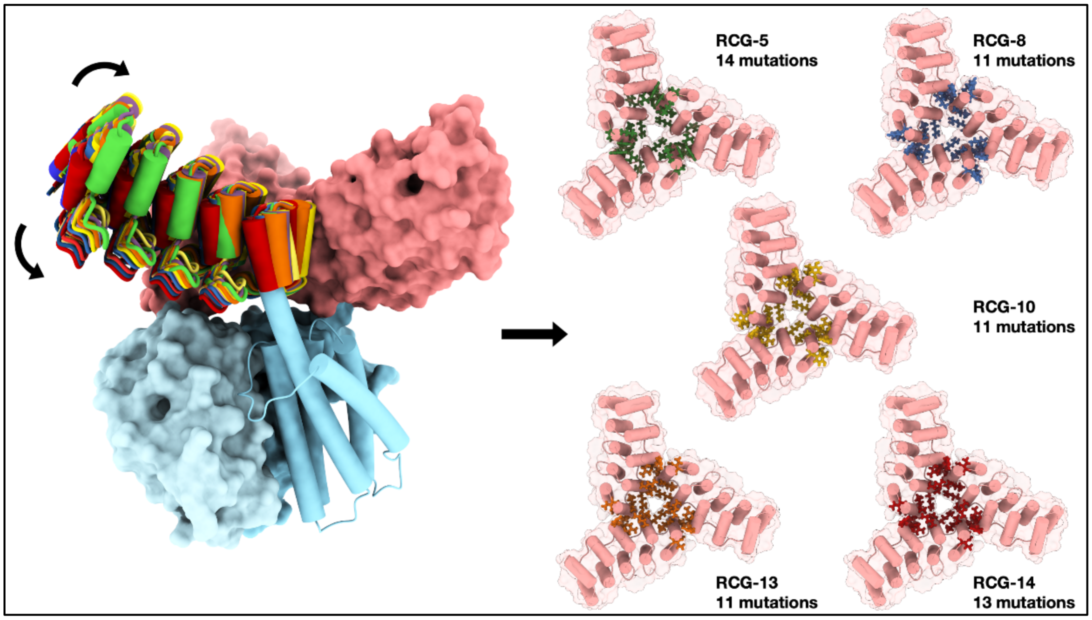

Figure S1. Rigidification of the designed scaffold by computational interface design between contacting DARPins. (left) Three protruding DARPins are shown fused to their protein cage subunits (blue) by a continuous alpha helical linker. A limited degree of natural flexibility (*i.e.* deviation from ideal helical parameters) was modeled in the alpha helix in order to generate distinct backbone models that sample slightly different modes of association in the new interface. One of the DARPins, subject to flexible modeling, is shown by multicolor models; the other two contacting DARPins are shown in salmon. Among top-scoring models from computational sequence design, five candidates (right) were found suitable for evaluation by cryo-EM. Mutated residues are shown in stick representation for each of the DARPins in a trimeric bundle.

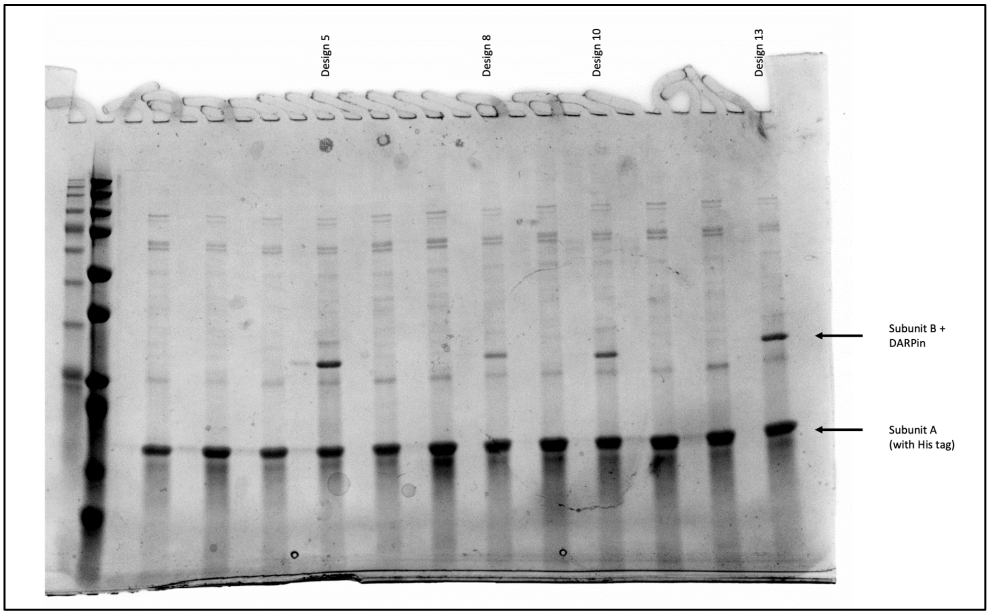

Figure S2. SDS PAGE gel showing co-elution of the two protein chains, A and B, comprising a scaffold. Subunit A (a component of the cage core) is His-tagged. Subunit B is a fusion between a cage core component and a DARPin that serves to bind diverse cargo proteins for imaging.

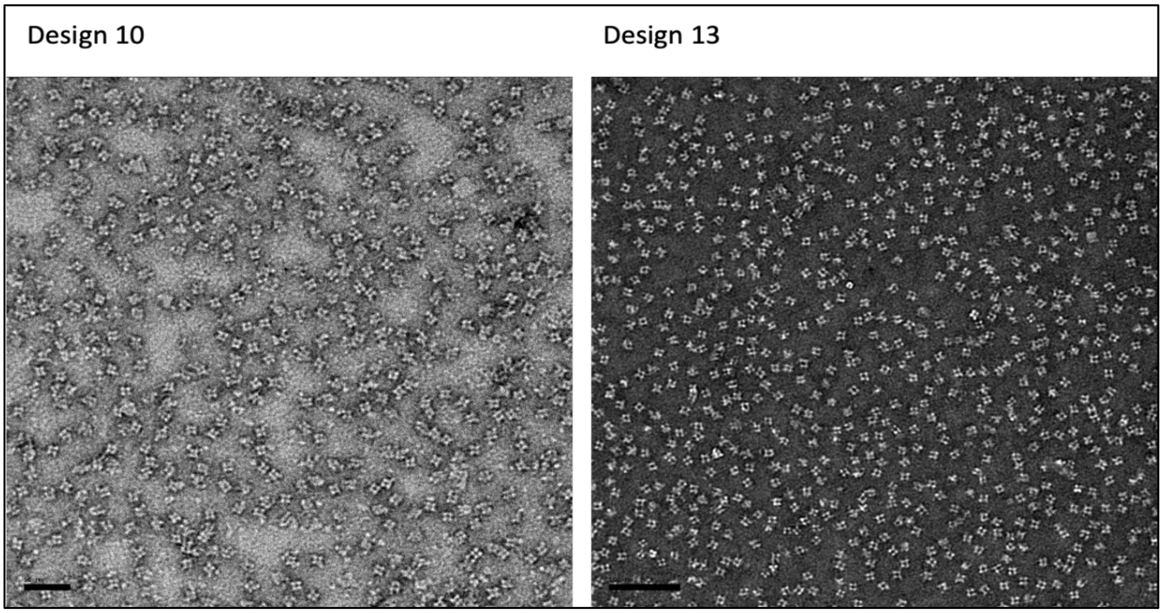

Figure S3. Negative stained electron micrographs of the rigidified imaging scaffold particles.

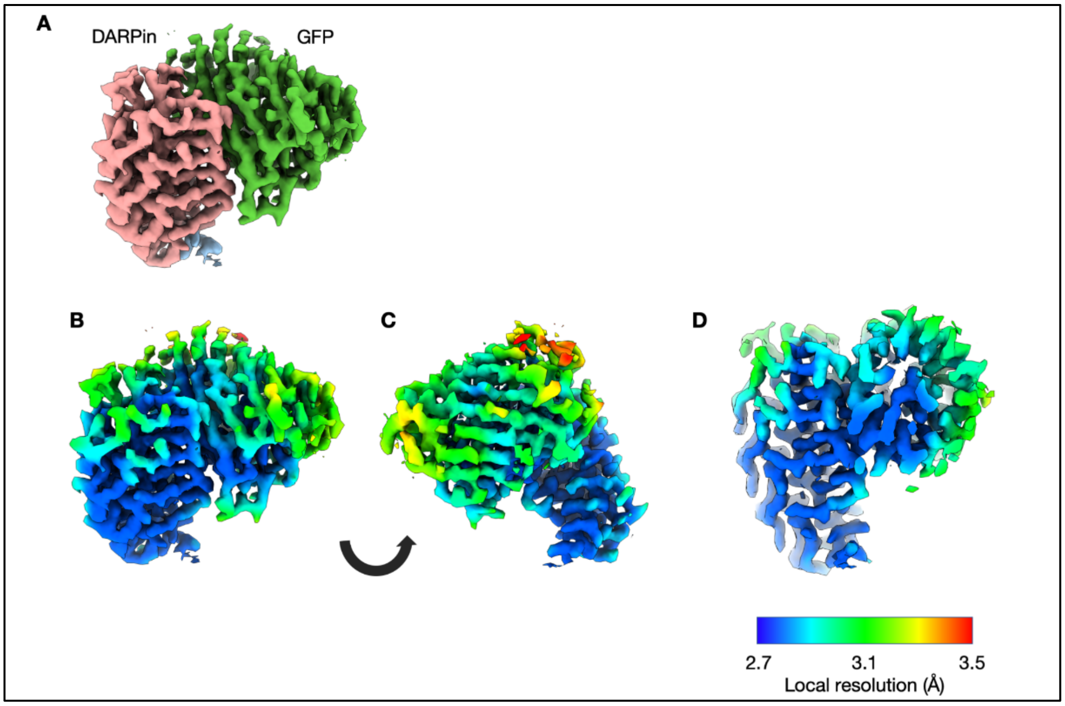

Figure S4. Local resolution for the focus-refined map of the GFP and DARPin. A. DARPin (salmon) and GFP (green). B and C, two rotated views of the map colored by local resolution. D, a cross section of the map.

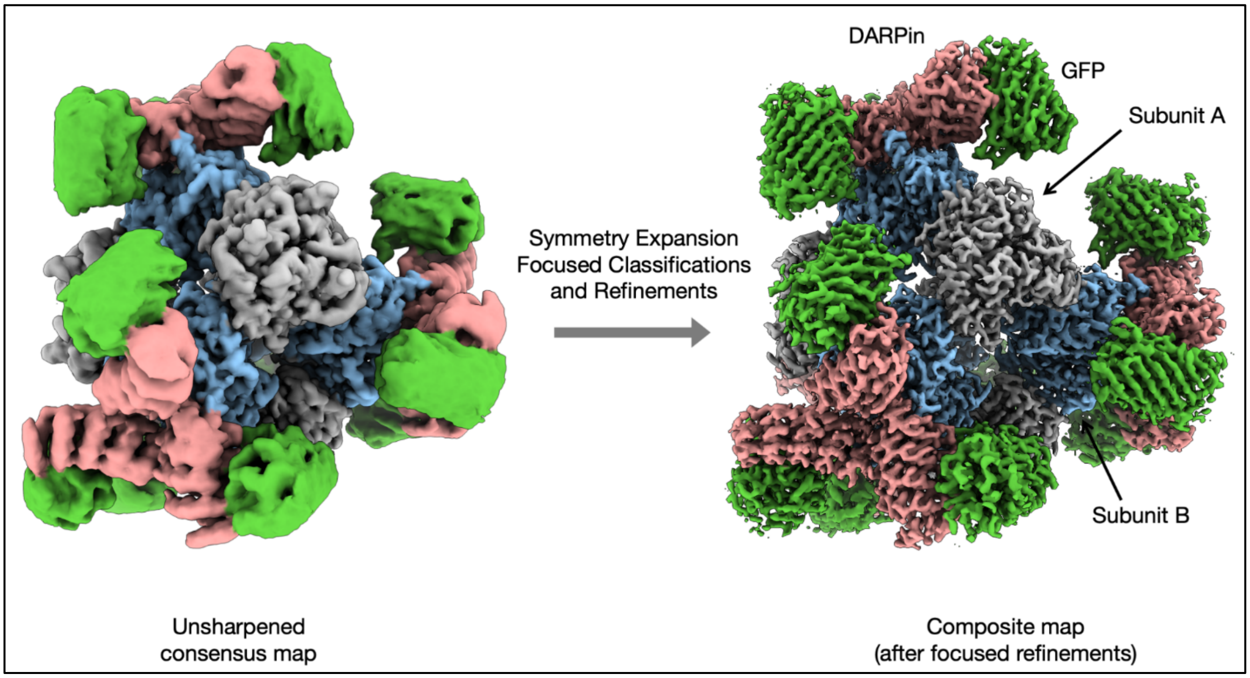

Figure S5. Cryo-EM densities for the imaging scaffold bound to GFP. The overall reconstruction (left) and a composite map of the focused refinements (right). After symmetry expansion (symmetry T), focused classifications and refinements were performed with a mask encompassing mainly one GFP (green) and one DARPin (salmon).

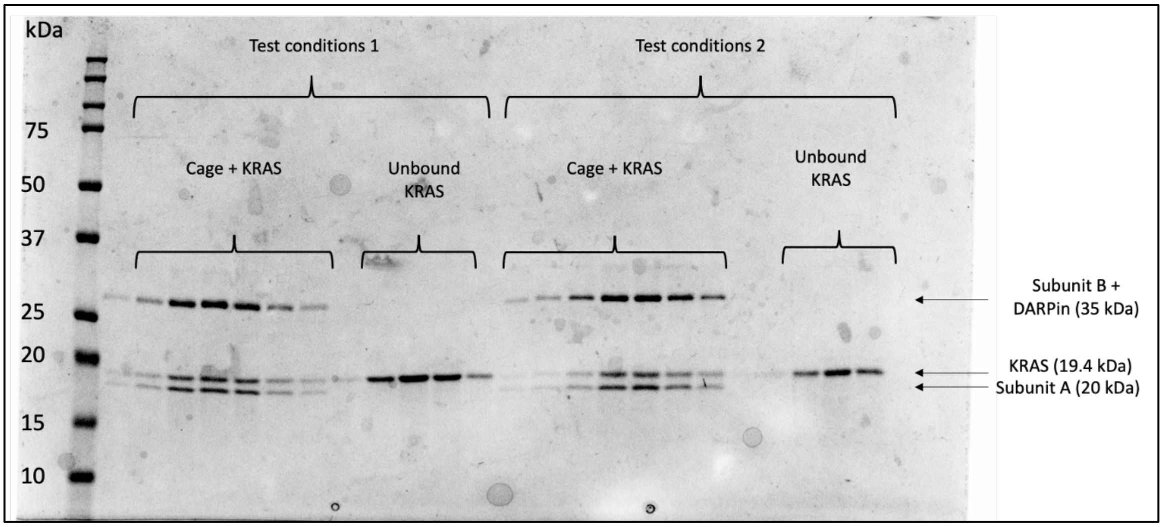

Figure S6. Binding assay of KRAS G13C to rigidified imaging scaffolds. The early SEC fractions show co-elution of the cage components with the KRAS (bound). The later fractions correspond to unbound KRAS. The higher band (~35 kDa) corresponds to the cage subunit B fused to the DARPin, the middle band corresponds to the KRAS protein (19.4 kDa) and the lower band is the cage subunit A (20kDa). Cage subunit A runs slightly smaller than its known size.

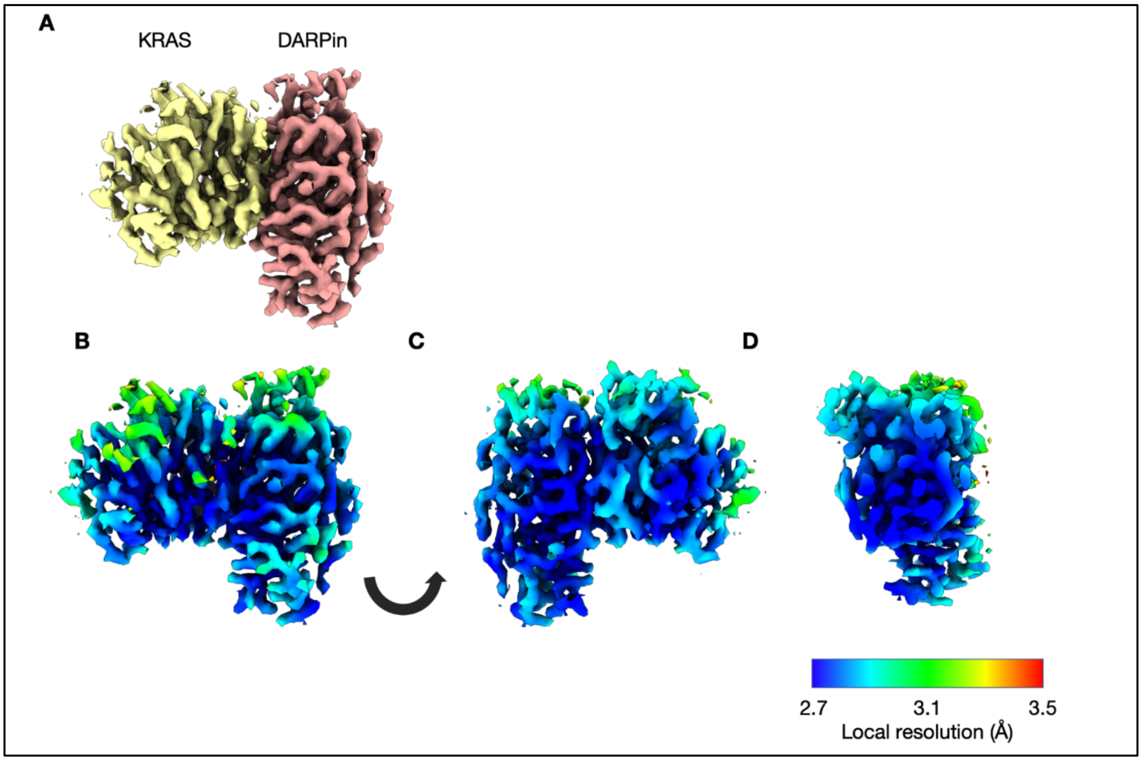

Figure S7. Local resolution for the focus-refined map of the KRAS G13C and DARPin. A. DARPin (salmon) and KRAS (yellow). B and C, two rotated views of the map colored by local resolution. D, cross section of the map.

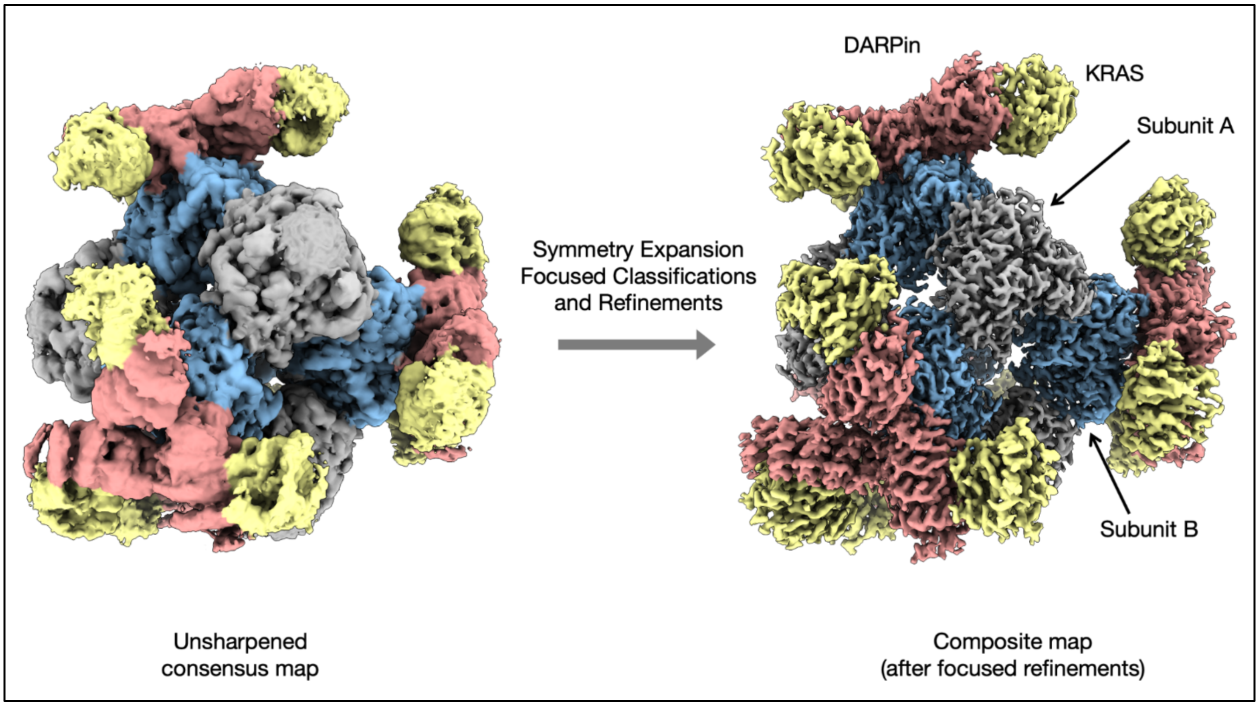

Figure S8. Cryo-EM densities for the imaging scaffold bound to KRAS G13C. The overall reconstruction (left) and a composite map of the focused refinements (right). After symmetry expansion (symmetry T), focused classifications and refinements were performed with a mask encompassing mainly one KRAS (yellow) and one DARPin (salmon).

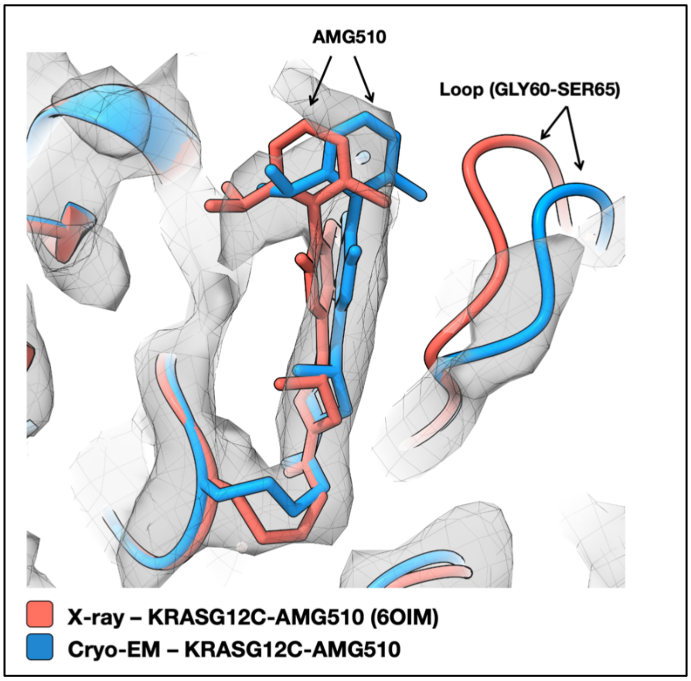

Figure S9. Binding position of the AMG510 drug to KRAS G12C in the cryo-EM structure reported here, compared to an x-ray crystal structure of the same complex. The cryo-EM density is shown with the refined cryo-EM model in blue. An earlier, high resolution x-ray crystal structure (pdb 6oim) is shown in salmon. [The comparison is shown after overlapping the protein backbone structures from the two coordinate sets.] The Q-score (i.e. agreement between atomic model and density map) for the terminal part of the drug molecule refined in the cryo-EM map is 0.59. For comparison, the Q-score for the drug molecule in the X-ray position compared to the cryo-EM map is considerably lower, 0.45. The refined conformation for the cryo-EM model is a better fit compared to the conformation see in the X-ray crystal structure.

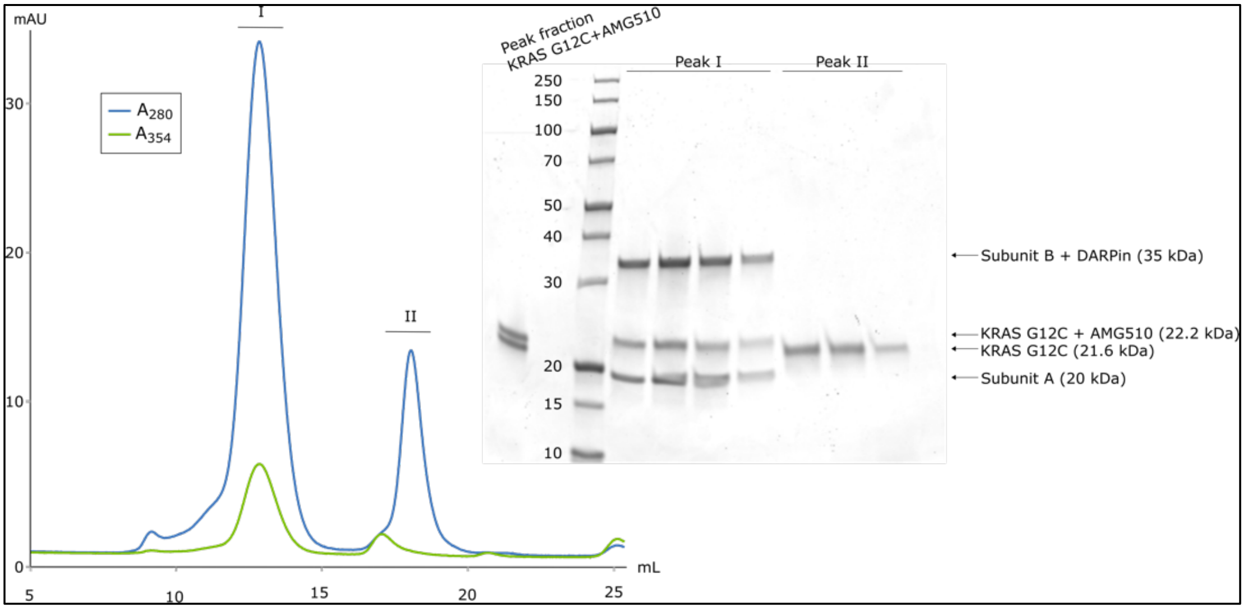

Figure S10. Imaging scaffold binding to KRAS G12C-AMG510. AMG510 shows maximum absorption at λ_{max}=354 nm, allowing for validation of complex formation between imaging scaffold and AMG510-bound KRAS. Covalent attachment of AMG510 to KRAS G12C increases the molecular weight by 560 Da which can be resolved on SDS-PAGE (inset). The SEC profile shows preferential binding of AMG510-bound KRAS G12C over inhibitor-free KRAS G12C to the imaging scaffold.

GFP (EMDB:29178)

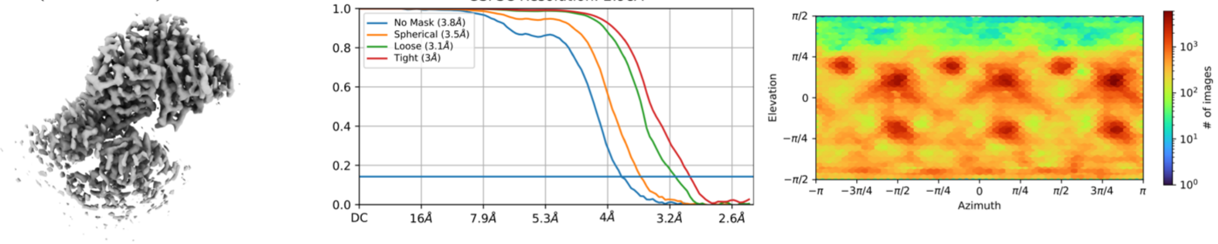

KRAS G13C (EMDB:29720)

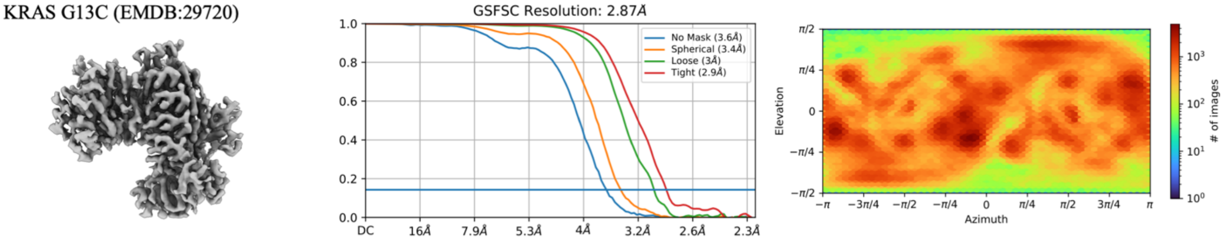

KRAS G12V (EMDB:29719)

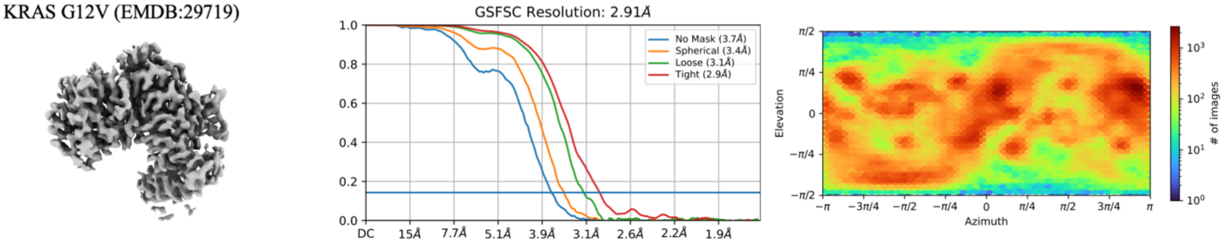

Scaffold cage core (EMDB:29700)

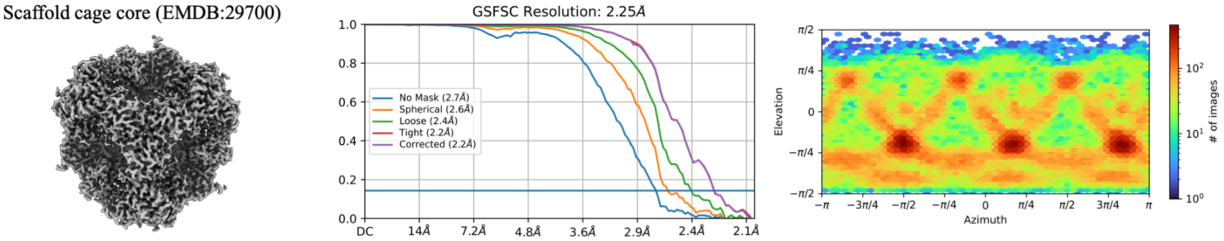

KRAS G12C (EMDB:29713)

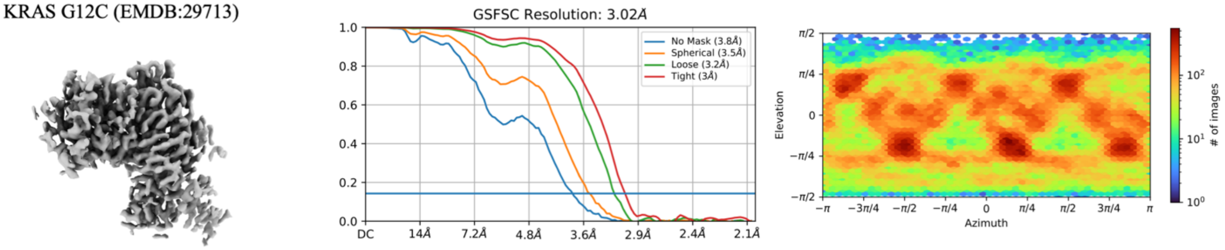

KRAS G12C AMG 510 (EMDB:29715)

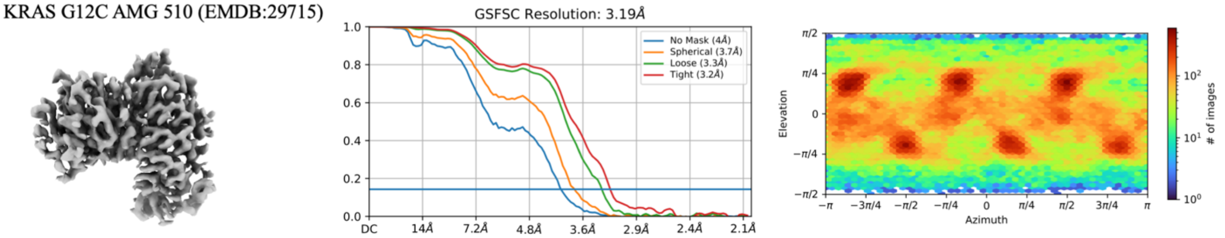

Figure S11. Cryo-EM maps summary. Cryo-EM maps reported in this manuscript with corresponding plots reporting the resolutions via gold standard FSC at a cutoff of 0.143 and the angular distribution of particles. The corrected FSC from EMDB:29700 is the FSC curve calculated using the tight mask with correction by noise substitution, provided when using the Homogeneous Refinement tool in cryoSPARC. Particle orientation density plots are shown in the right column.

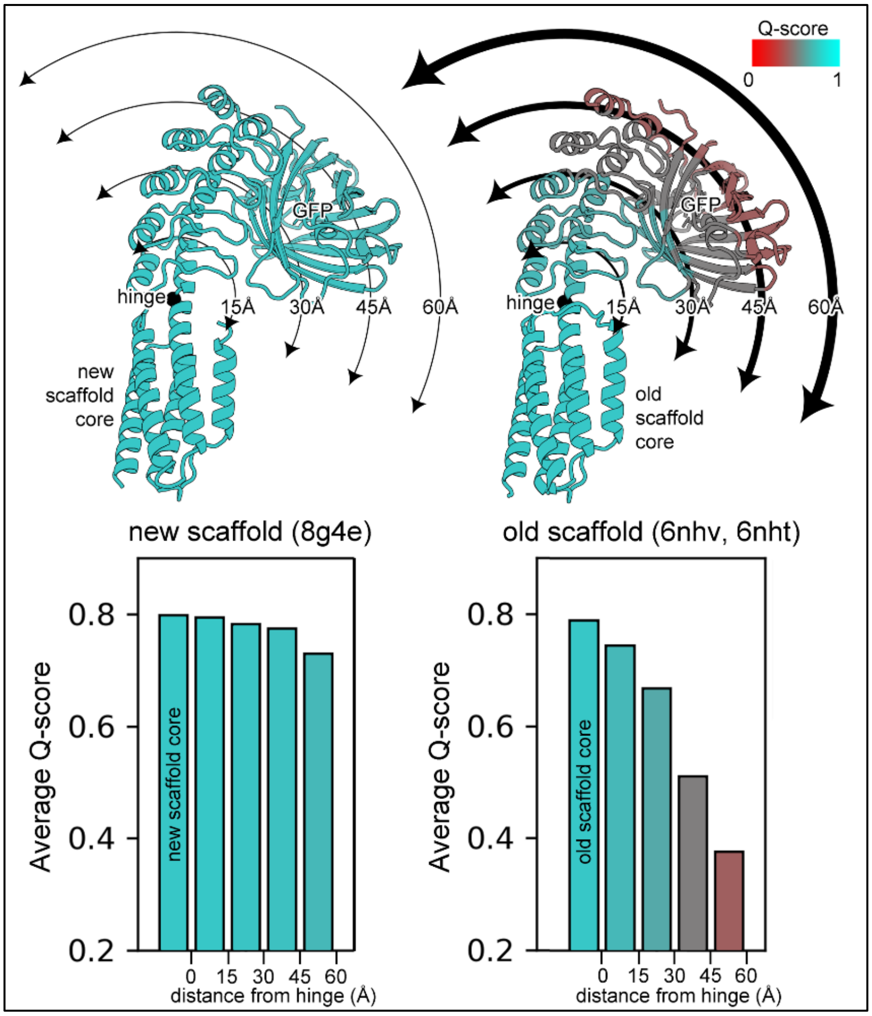

Figure S12. Improvements in Q-score values (i.e. correlation between atomic model and density map) are observed for the new rigidified imaging scaffolds (left) when compared with the old imaging scaffolds (right). The old scaffold exhibits a distribution of Q-scores that increases dramatically as a function of distance from the hinge point. The new scaffold largely eliminates this dependence, showing that elimination of the hinge motion is largely responsible for the improved quality of the resulting density map.

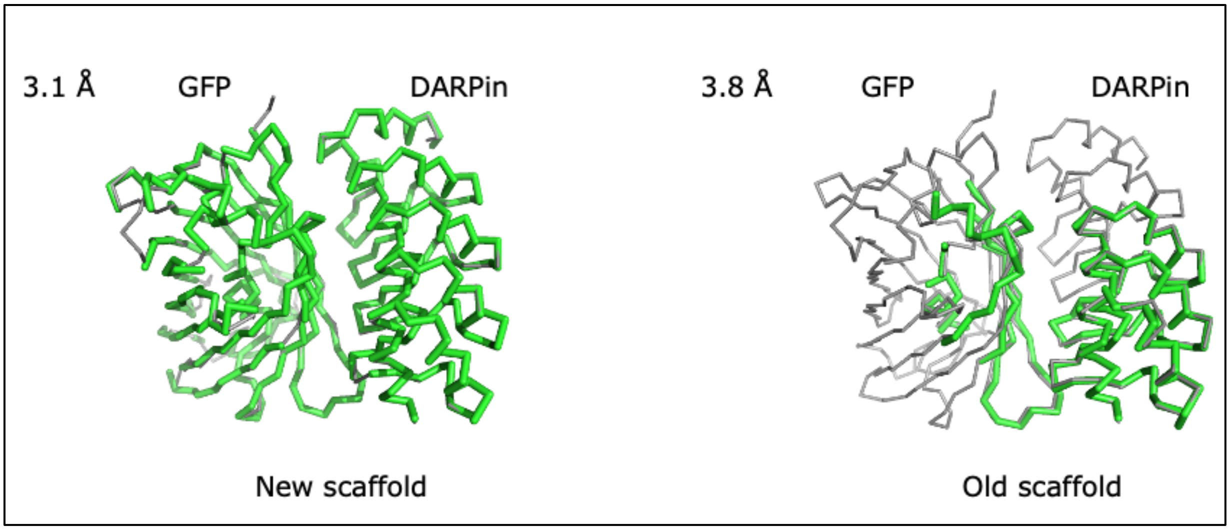

Figure S13. Comparison of automatic atomic model-building trials using density maps from the present scaffold vs a prior scaffold prior to engineering for rigidification. For the new scaffold, 95% of the GFP scaffold could be correctly built automatically, compared to 28% for the map obtained from the prior scaffold. The models automatically built by the program ModelAngelo are shown in green, overlaid with the final structure in gray.

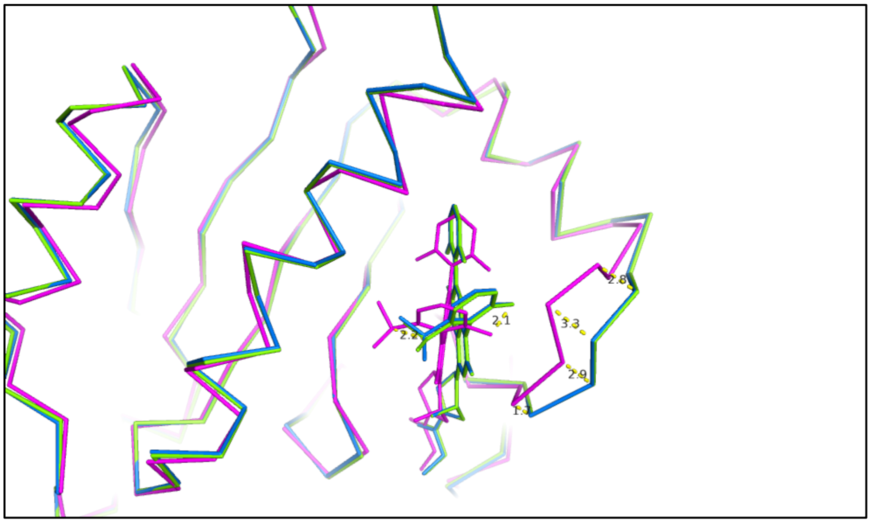

Figure S14. Comparison of models at the drug binding site after refinement against independent half-maps – i.e. maps obtained by 3-D reconstructions from independent sets of cryo-EM projection images. Model refined with half map A (green), half map B (marine), and the crystal structure 6oim (magenta). The coordinate differences between the two half map-refined models are approximately 1/10 the magnitude of the differences between the crystal structure (6oim) and the scaffolded cryo-EM structure (8g47).

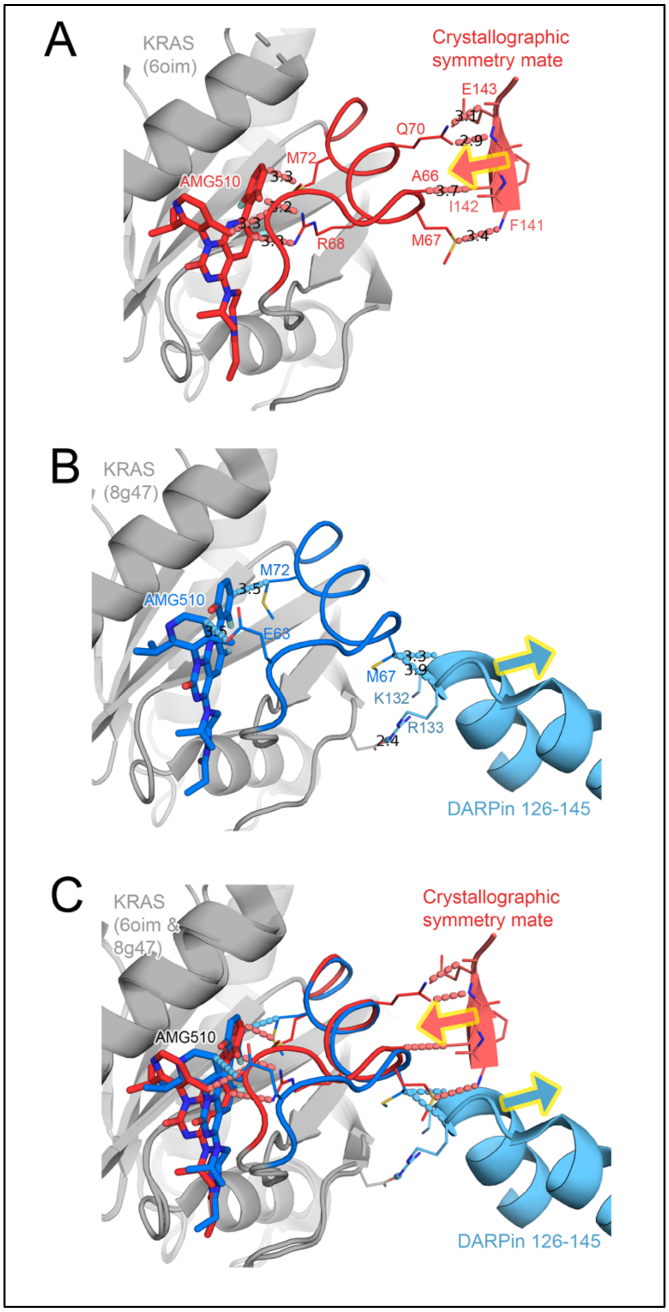

Figure S15. Potential influence of protein packing forces on the conformation of the AMG510 drug molecule and the neighboring binding pocket on KRAS. (A) Crystal structure of KRAS-AMG510 (6oim). Gray colors indicate residues structurally conserved with the new scaffolded cryo-EM KRAS structure (8g47). Red indicates structurally variable residues. Pink indicates a crystallographically related symmetry copy of the KRAS molecule. The neighboring molecule impinges on a helix (red) that contacts AMG510. (B) Scaffolded cryo-EM structure of KRAS-AMG510 (8g47). Residues that differ in structure from 6oim are shown in deep blue color. Light blue indicates a segment of DARPin used to display KRAS. (C) Superposition with arrows showing the different angles at which packing forces influence the AMG510 binding site in the two structures.

A

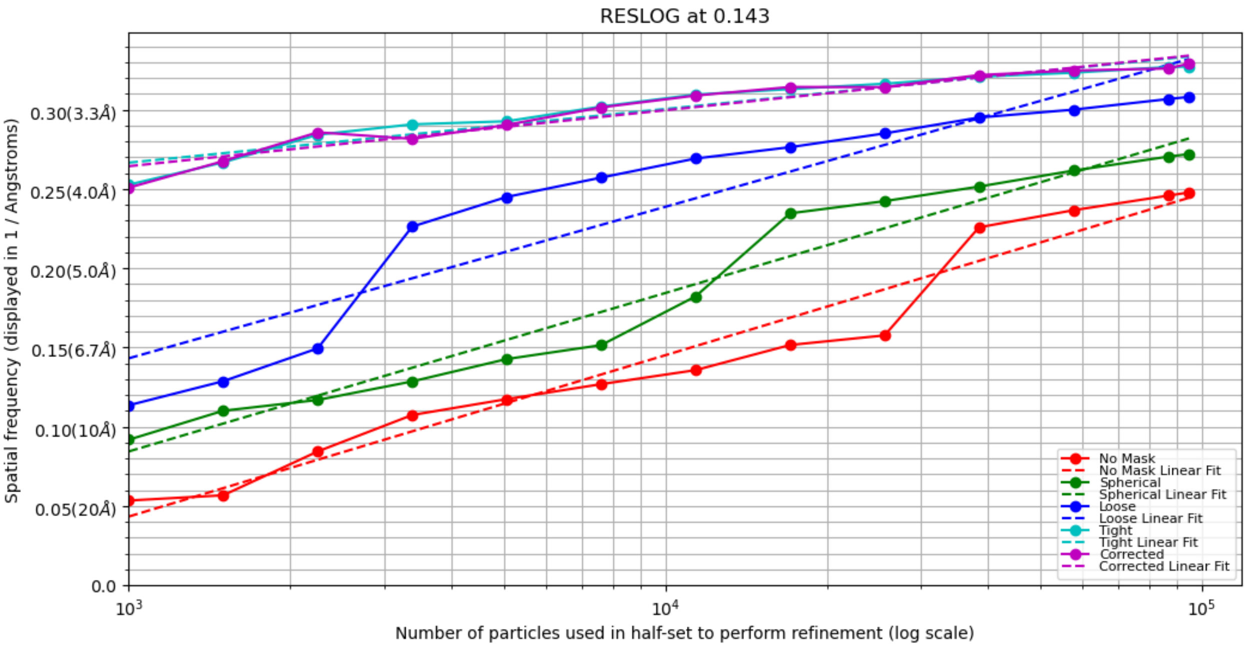
RESLOG at 0.143
Spatial frequency (displayed in 1 / Angstroms)
0.30(3.3Å)
0.25(4.0Å)
0.20(5.0Å)
0.15(6.7Å)
0.10(10Å)
0.05(20Å)
0.0
10^3
10^4
10^5
Number of particles used in half-set to perform refinement (log scale)
No Mask
No Mask Linear Fit
Spherical
Spherical Linear Fit
Loose
Loose Linear Fit
Tight
Tight Linear Fit
Corrected
Corrected Linear Fit

B

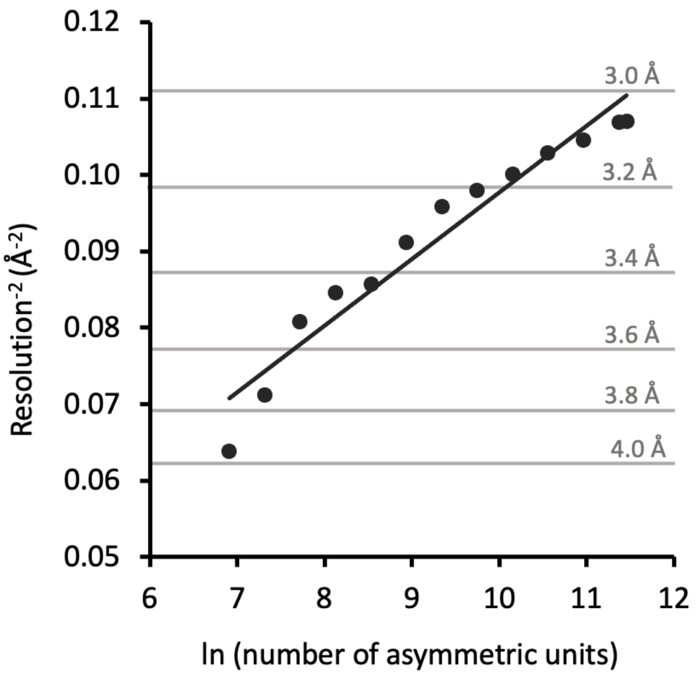
Resolution^{-2} (Å^{-2})
0.12
0.11
0.10
0.09
0.08
0.07
0.06
0.05
3.0 Å
3.2 Å
3.4 Å
3.6 Å
3.8 Å
4.0 Å
6
7
8
9
10
11
12
ln (number of asymmetric units)

C

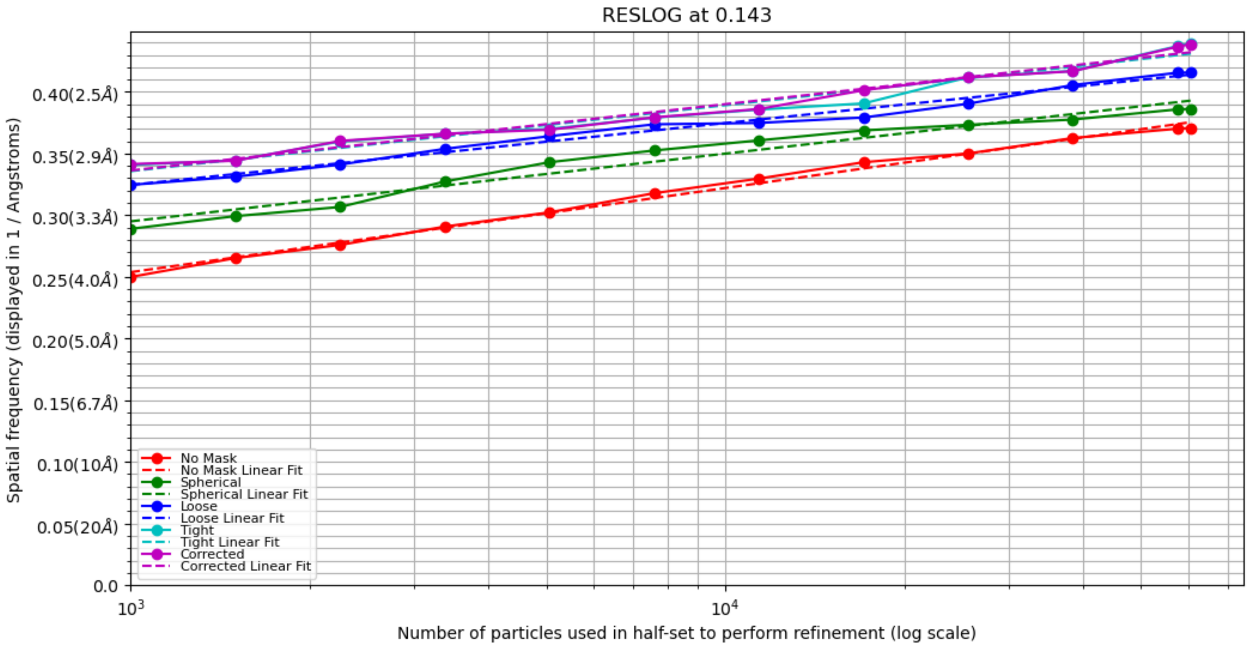

D

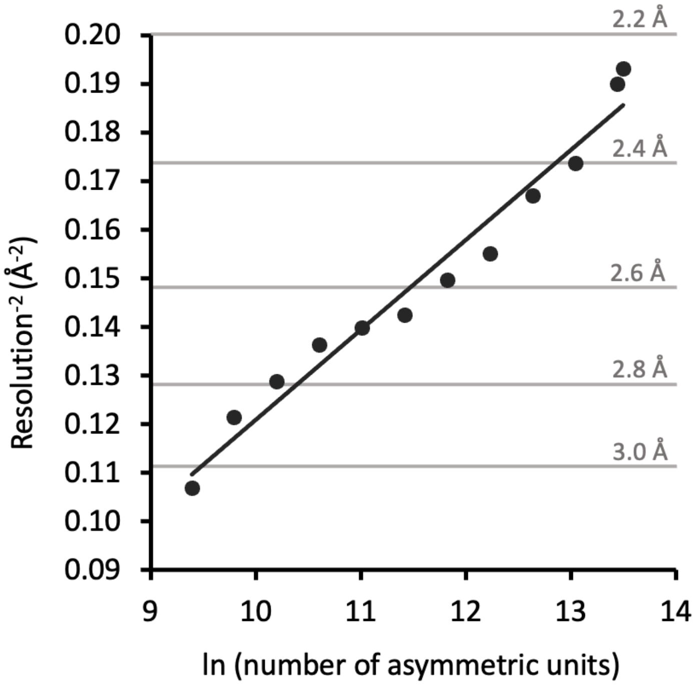

Figure S16. Plots of dependence of estimated resolution on number of particles based on the ResLog program (1) calculated using (a, b) the particles used for the reconstructions of KRAS G12C (C1 symmetry, emdb 29713) and (c, d) the particles used for the reconstruction of the core of the imaging scaffold with KRAS G12C (T symmetry, emdb 29700).

Supplementary text:

Recent cryo-EM scaffolding results:

In concurrent developments using antibody/nanobody approaches, the resolutions reported have been better for larger cargo proteins – e.g. 2.49 Å for a ~200 kDa receptor complex (2); 2.8 Å for a 64 kDA protein (3), 3.03 Å for a 58 kDa protein (4); ~3.2 Å for a 52 kDa protein (5), 3.47 Å and 3.78 Å for ~50 kDa proteins using NabFabs (6). For proteins smaller than 50 kDa, the finest resolution so far are 3.0−4.0 Å for a 11 kDa KIX domain fused to apoferritin (7) and roughly 3.2 Å for a 23 kDa protein bound to a scaffold ensemble (8). An overall resolution of 3.2 Å was reported for the overall complex between the scaffold and KDELR; a resolution range of 3.0 to 3.5 Å was estimated for the just the KDELR protein. These are summarized in Table S3.

Protein sequences

RCG-1, cage protein T33-51, Subunit A:
MRITTKVGDKGSTRLFGGEEVWKDDPIIEANGTLDELTSFIGEAKHYVDEEMKGILEEIQ
NDIYKIMGEIGSKGKIEGISEERIKWLAGLIERYSEMVNKLSFVLPGGTLESAKLDVCRTI
ARRAERKVATVLREFGIGTLAAIYLALLSRLLFLLARVIEIEKNKLKEVRSHHHHHH

RCG-5, Subunit B, which binds GFP:
MFTRRGDQGETDLANRARVGKDSPVVEVQGTIDELNSFIGYALVLSRWDDIRNDLFRIQ
NDLFVLGEDVSTGGKGRTVTMDMIIYLIKRSVEMKAEIGKIELFVVPGGSVESASLHMA
RAVSRRLERRIKAASELTEINANVLLYANMLSNILFMHALISNKRKEELDKKLLEAARAG
DKYAVDALLAKGADVNAADDVGVTPLHLAAQRGHLEIVEVLLKRGWDINAADLWGQ
TPLHLAATAGHLEIVELLLWYGADVNARDNIGHTPLHLAAWAGHLEIVEVLLKYGADV
NAQDKFGKTPFDLAIDNGNEDIAEVLQKAA

RCG-8, Subunit B, which binds GFP:
MFTRRGDQGETDLANRARVGKDSPVVEVQGTIDELNSFIGYALVLSRWDDIRNDLFRIQ
NDLFVLGEDVSTGGKGRTVTMDMIIYLIKRSVEMKAEIGKIELFVVPGGSVESASLHMA
RAVSRRLERRIKAASELTEINANVLLYANMLSNILFMHALISNKRKEELDKKLLEAARAG
YDDKVAWLLALGADVNAADDVGVTPLHLAAQRGHLEIVEVLLKRGADINAADLWGQ
TPLHLAATAGHLEIVEKLLRCGADVNARDNIGHTPLHLAAWAGHLEIVEVLLKYGADV
NAQDKFGKTPFDLAIDNGNEDIAEVLQKAA

RCG-10, Subunit B, which binds GFP:
MFTRRGDQGETDLANRARVGKDSPVVEVQGTIDELNSFIGYALVLSRWDDIRNDLFRIQ
NDLFVLGEDVSTGGKGRTVTMDMIIYLIKRSVEMKAEIGKIELFVVPGGSVESASLHMA
RAVSRRLERRIKAASELTEINANVLLYANMLSNILFMHALISNKRKEELDKKLLEAARAG
YDDQVAALLAKGADVNAADDVGVTPLHLAAQRGHLEIVEVLLKRGADINAADLWGQT
PLHLAATAGHLEIVELLLRWGADVNARDNIGHTPLHLAAWAGHLEIVEVLLKYGADVN
AQDKFGKTPFDLAIDNGNEDIAEVLQKAA

RCG-13, Subunit B, which binds GFP:
MFTRRGDQGETDLANRARVGKDSPVVEVQGTIDELNSFIGYALVLSRWDDIRNDLFRIQ
NDLFVLGEDVSTGGKGRTVTMDMIIYLIKRSVEMKAEIGKIELFVVPGGSVESASLHMA
RAVSRRLERRIKAASELTEINANVLLYANMLSNILFMHALISNKRKDELDKKLLEAARA
GIDDAVAALLAKGADVNAADDVGVTPLHLAAQRGHLEIVKVLLLRGADINAADLWGQ
TPLHLAATAGHLEIVELLLRCGADVNARDNIGHTPLHLAAWAGHLEIVEVLLKYGADV
NAQDKFGKTPFDLAIDNGNEDIAEVLQKAA

RCG-14, Subunit B, which binds GFP:
MFTRRGDQGETDLANRARVGKDSPVVEVQGTIDELNSFIGYALVLSRWDDIRNDLFRIQ
NDLFVLGEDVSTGGKGRTVTMDMIIYLIKRSVEMKAEIGKIELFVVPGGSVESASLHMA
RAVSRRLERRIKAASELTEINANVLLYANMLSNILFMHALISNKRRDERNKKLLEAARA
GIDDAVDWLLALGADVNAADDVGVTPLHLAAQRGHLEIVKVLLSRGADINAADLWGQ
TPLHLAATAGHLEIVELLLRCGADVNARDNIGHTPLHLAAWAGHLEIVEVLLKYGADV
NAQDKFGKTPFDLAIDNGNEDIAEVLQKAA
RCG-33, Subunit B, which binds KRAS:
MFTRRGDQGETDLANRARVGKDSPVVEVQGTIDELNSFIGYALVLSRWDDIRNDLFRIQ
NDLFVLGEDVSTGGKGRTVTMDMIIYLIKRSVEMKAEIGKIELFVVPGGSVESASLHMA
RAVSRRLERRIKAASELTEINANVLLYANMLSNILFMHALISNKRKEELDKKLLEAARAG
QDDEVAALLAKGADVNAHDTFGFTPLHLAALYGHLEIVEVLLKRGADINADDSYGRTP
LHLAAMRGHLEIVELLLRWGADVNAADEEGRTPLHLAAKRGHLEIVEVLLKNGADVN
AQDKFGKTAFDISIDNGNEDLAEILQKL

Super folder GFP V206A:
MSKGEELFTGVVPILVELDGDVNGHKFSVRGEGEGDATNGKLTLKFICTTGKLPVPWPT
LVTTLTYGVQCFSRYPDHMKRHDFFKSAMPEGYVQERTISFKDDGTYKTRAEVKFEGD
TLVNRIELKGIDFKEDGNILGHKLEYNFNSHNVYITADKQKNGIKANFKIRHNVEDGSVQ
LADHYQQNTPIGDGPVLLPDNHYLSTQSALSKDPNEKRDHMVLLEFVTAAGITHHHHH
H

KRAS-G13C:
GSMTEYKLVVVGAGCVGKSALTIQLIQNHFVDEYDPTIEDSYRKQVVIDGETSLLDILDT
AGQEEYSAMRDQYMRTGEGFLLVFAINNTKSFEDIHHYREQIKRVKDSEDVPMVLVGN
KSDLPSRTVDTKQAQDLARSYGIPFIETSAKTRQGVDDAFYTLVREIRKHKEK

KRAS-G12V:
GSHMTEYKLVVVGAVGVGKSALTIQLIQNHFVDEYDPTIEDSYRKQVVIDGETCLLDIL
DTAGQEEYSAMRDQYMRTGEGFLCVFAINNTKSFEDIHHYREQIKRVKDSEDVPMVLV
GNKCDLPSRTVDTKQAQDLARSYGIPFIETSAKTRQGVDDAFYTLVREIRKH

KRAS-G12C:
MGSSHHHHHHSSGENLYFQSMTEYKLVVVGACGVGKSALTIQLIQNHFVDEYDPTIEDS
YRKQVVIDGETCLLDILDTAGQEEYSAMRDQYMRTGEGFLCVFAINNTKSFEDIHHYRE
QIKRVKDSEDVPMVLVGNKCDLPSRTVDTKQAQDLARSYGIPFIETSAKTRQGVDDAFY
TLVREIRKHKEK

Table S1. Cryo-EM data collection, image analysis, modeling, refinement, and validation statistics

**Dataset**	**GFP + imaging scaffold (RCG-1, RCG-10)**	**KRAS-G13C + imaging scaffold (RCG-1, RCG-33)**	**KRAS-G12V + imaging scaffold (RCG-1, RCG-33)**	**KRAS-G12C + imaging scaffold (RCG-1, RCG-33)**	**KRAS-G12C + imaging scaffold (RCG-1, RCG-33)**	**KRAS-G12C-AMG510 + imaging scaffold (RCG-1, RCG-33)**
**Map refinement**	Focused on GFP + DARPin domain	Focused on KRAS-G13C + DARPin domain	Focused on KRAS-G12V + DARPin domain	Global	Focused on KRAS-G12C + DARPin domain	Focused on KRAS-G12C-AMG510 + DARPin domain
**Data collection and processing**						
Microscope	Titan Krios	Titan Krios	Titan Krios	Titan Krios	Titan Krios	Titan Krios
Voltage (keV)	300	300	300	300	300	300
Detector	K3 Summit	K3 Summit	K3 Summit	Falcon4	Falcon4	Falcon4
Nominal magnification	81,000	81,000	105,000	155,000	155,000	155,000
Data Acquisition Software	SerialEM	SerialEM	SerialEM	EPU	EPU	EPU
Electron dose ($e^-/Å^2$)	33	40	48	40	40	40
Pixel Size (Å)	1.1	1.1	0.856	0.5	0.5	0.5
Defocus range (µm)	-1.00 to -2.20	-1.00 to -2.20	-1.00 to -2.20	-1.00 to -2.25	-1.00 to -2.25	-1.00 to -2.25
Number of movies (#)	3,575	2,000	3,616	7,312	7,312	2,072
Number of particles	1,221,977	1,653,856	685,177	121,441	189,266	171,436
Symmetry imposed	C1	C1	C1	T	C1	C1
Resolution (Å)	3.0	2.9	2.9	2.2	3.0	3.2
FSC threshold	0.143	0.143	0.143	0.143	0.143	0.143
**Refinement**						
Initial model used (PDB code)	*de novo*	*de novo*	KRAS-G13C	5cy5	5o2s	6oim
Non-hydrogen atoms	3075	2504	2529	2244	2498	2575
Protein residues	396	320	323	285	323	325
Residue range modelled	GFP (1-231) + RCG-10 (155-322)	KRAS-G13C (1-168) + RCG-33 (169-320)	KRAS-G12V (1-165) + RCG-33 (163-320)	RCG-1 (24-166) + RCG-33 (23-171)	KRAS-G12C (1-165) + RCG-33 (163-320)	KRAS-G12C-AMG510 (1-168) + RCG-33 (163-319)
R.M.S. deviations						
Bond lengths (Å)	0.002	0.002	0.002	0.002	0.003	0.002
Bond angles (°)	0.45	0.45	0.44	0.36	0.46	0.44
**Validation**						
MolProbity score	1.72	1.47	1.37	1.14	1.49	1.45
Clashscore	7.3	5.4	3.7	3.5	8.0	8.3
Poor rotamers (%)	2.2	1.5	1.9	0.0	1.0	0.4
Ramachandran (%)						
Favored	97.7	97.8	98.8	99.3	97.8	99.1
Allowed	2.3	2.2	1.2	0.7	2.2	0.9
Disallowed	0.0	0.0	0.0	0.0	0.0	0.0
Fit to map (CC_{mask})	0.8176	0.7961	0.8436	0.8216	0.7607	0.7157
**Accession codes**						
EMDB (maps)	29718	29720	29719	29700	29713	29715
PDB (model)	8G4E	8G4H	8G4F	8G3K	8G42	8G47

Table S2. Rmsd alpha-carbons (Å) values between X-ray structures and the cryo-EM maps.

Target (pdb ID)	Moving	RMSD alpha-carbons (Å)	Number of atom pairs superimposed
6oim	8G42-kras-g12c-gdp	1.08	161
6oim	8G47-kras-g12c-AMG510	0.73	161
6oim	8G4F-kras-g12v-gdp	0.99	161
6oim	8G4H-kras-g13c-gdp	1.07	161
6b9c	8G4E-gfp.pdb	0.59	202

Table S3. Comparison of size and resolution for contemporary Cryo-EM protein fiducial/scaffolds including their binders and targets.

Citation	Total scaffold size(s) (kDa)	Modular binder - size (kDa)	Protein Target - size(s) (kDa)	Reported Resolution (Å)
Liu, Y. et al. 2018	591.5	DARPin - 16.8	GFP - 26	3.8
Uchański, T. et al. 2021	56, 100	Nanobody - 14	WbaP-B - 114<br>GABAaR - 288.5 pentamer (57.7 monomer)	~5<br>2.49
Block, J. S. et al. 2021	76.7	Nanobody - 13.3	VcNorM - 49.4<br>ScaDMT - 49.3	3.47<br>3.78
Wu and Rappaport 2021	120	Nanobody - 15	SARS-CoV2 spike RBD - 22<br>KDEL receptor - 23	3.6<br>3.2
Zhang, K. et al. 2022	291.7	N/A	KIX - 11.4	3-4
This work	664.5	DARPin - 17.2	KRAS - 19.4<br>GFP - 26	2.9<br>3.1

References:

1. S. M. Stagg, A. J. Noble, M. Spilman, M. S. Chapman, ResLog plots as an empirical metric of the quality of cryo-EM reconstructions. *Journal of Structural Biology* **185**, 418–426 (2014).

2. T. Uchański, *et al.*, Megabodies expand the nanobody toolkit for protein structure determination by single-particle cryo-EM. *Nat Methods* **18**, 60–68 (2021).

3. M. A. Herzik, M. Wu, G. C. Lander, High-resolution structure determination of sub-100 kDa complexes using conventional cryo-EM. *Nature Communications* **10**, 1032 (2019).

4. R. J. Cater, *et al.*, Structural basis of omega-3 fatty acid transport across the blood–brain barrier. *Nature* **595**, 315–319 (2021).

5. X. Fan, *et al.*, Single particle cryo-EM reconstruction of 52 kDa streptavidin at 3.2 Angstrom resolution. *Nat Commun* **10**, 2386 (2019).

6. J. S. Bloch, *et al.*, Development of a universal nanobody-binding Fab module for fiducial-assisted cryo-EM studies of membrane proteins. *Proceedings of the National Academy of Sciences* **118**, e2115435118 (2021).

7. K. Zhang, *et al.*, Cryo-EM, Protein Engineering, and Simulation Enable the Development of Peptide Therapeutics against Acute Myeloid Leukemia. *ACS Central Science* **8**, 214–222 (2022).

8. X. Wu, T. A. Rapoport, Cryo-EM structure determination of small proteins by nanobody-binding scaffolds (Legobodies). *Proceedings of the National Academy of Sciences* **118**, e2115001118 (2021).
